# Gut microbiome diversity, variability, and latent community types compared with shifts in body weight during the freshman year of college in dormitory-housed adolescents

**DOI:** 10.1080/19490976.2023.2250482

**Published:** 2023-08-29

**Authors:** Alex E. Mohr, Mary M. Ahern, Dorothy D. Sears, Meg Bruening, Corrie M. Whisner

**Affiliations:** a College of Health Solutions, Arizona State University, Phoenix, AZ, USA; b Department of Nutritional Sciences, College of Health and Human Development, Pennsylvania State University, University Park, PA, USA; c Center for Health Through Microbiomes, Biodesign Institute, Arizona State University, Tempe, AZ, USA

**Keywords:** Microbiota, stability, *Prevotella*, *Bacteroides*, state transition, Dirichlet allocation, obesity, adolescent, college, diet

## Abstract

Significant human gut microbiome changes during adolescence suggest that microbial community evolution occurs throughout important developmental periods including the transition to college, a typical life phase of weight gain. In this observational longitudinal study of 139 college freshmen living in on-campus dormitories, we tracked changes in the gut microbiome via 16S amplicon sequencing and body weight across a single academic year. Participants were grouped by weight change categories of gain (WG), loss (WL), and maintenance (WM). Upon assessment of the community structure, unweighted and weighted UniFrac metrics revealed significant shifts with substantial variation explained by individual effects within weight change categories. Genera that positively contributed to these associations with weight change included *Bacteroides*, *Blautia*, and *Bifidobacterium* in WG participants and *Prevotella* and *Faecalibacterium* in WL and WM participants. Moreover, the *Prevotella*/*Bacteroides* ratio was significantly different by weight change category, with WL participants displaying an increased ratio. Importantly, these genera did not display co-dominance nor ease of transition between *Prevotella*- and *Bacteroides*-dominated states. We further assessed the overall taxonomic variation, noting the increased stability of the WL compared to the WG microbiome. Finally, we found 30 latent community structures within the microbiome with significant associations with waist circumference, sleep, and dietary factors, with alcohol consumption chief among them. Our findings highlight the high level of individual variation and the importance of initial gut microbiome community structure in college students during a period of major lifestyle changes. Further work is needed to confirm these findings and explore mechanistic relationships between gut microbes and weight change in free-living individuals.

## Plain Language Summary

The freshman year of college is a transitional period that may provide insights into the relationship between the gut microbiome and body weight regulation due to the lifestyle changes that increase vulnerability to weight change. During this critical period many of the lifestyle factors that influence body weight formalize and have important bearing on health outcomes throughout an individual’s life. In this college-aged population, shifts in community structure and variability of gut microbes were different by weight change trajectory. Genera that underpinned these shifts such as *Bacteroides*, *Blautia*, *Bifidobacterium*, *Prevotella*, and *Faecalibacterium* displayed varying degrees of inter-individual variability and, in some instances, resistance to alternative states. Accounting for these considerations in the context of body weight control in adolescents may prove useful for improving target outcomes in an intervention setting.

## Introduction

Emerging adulthood is a formative developmental period, hallmarked by independence and substantial changes in living situation and lifestyle behaviors. As a group, incoming college students living in on-campus dormitories typify these factors. Changes in lifestyle behaviors are particularly evident for body weight changes during the first year as unhealthy eating behaviors and low levels of physical activity contribute to approximately 70% of student weight gain during the freshman year (~1–2 kg), often typified as excess weight gain.^1,2^ Such changes are problematic for health outcomes as young individuals with overweight and obesity are more likely to remain with overweight or obesity into adulthood.^3^

Differences in the assemblage of gut microbial communities have been broadly observed between individuals with obese and lean body types across different age ranges, including children and adolescents.^4,5^ These compositional differences remain significantly distinct even when controlling for constitutional factors like genetics.^6^ The community landscape of the gut microbiome tracks with a multitude of health features, forming a bi-directional relationship with the host. Notable moderating factors that drive this relationship include diet, physical activity, weight status, and age.^7–10^ Therefore, understanding the relationship between the complex community of gut microbes and health over the lifespan is important for improving outcomes, with a shifting focus from cross-sectional to longitudinal investigations. Results from animal models suggest that microbes influence weight gain, but this relationship in humans remains unclear.^11^ Indeed, clinical study challenges persist and host–microbiome relationships in humans have been difficult to examine in rigorous investigations.^12,13^ For instance, variance at the individual level (e.g., baseline microbiome composition and functional capacity) greatly confounds the interpretation of intervention or environmental exposure outcomes analyses across study populations.^14–16^ Differences in host features and gut microbial structure and function are important considerations, particularly when exploring the underpinnings of divergent health trajectories.

In late-stage adolescence as individuals emerge into adulthood, many lifestyle factors that influence excess body weight formalize and have important bearing on health outcomes throughout an individual’s life. Age has been identified as an important factor in gut microbiome diversity, with some reports demonstrating statistically significant differences between adolescents and adults.^17,18^ Animal studies suggest that adolescents have a malleable microbiome composition that responds more effectively than adults to lifestyle interventions and results in lasting changes in body composition.^19^ However, the implications of the relationship between age and community dynamics in the human gut microbiome on the health of adolescents remain relatively unchartered.

Limited longitudinal data are available in which shifts in the gut microbiome in free-living populations can be examined during periods of expected weight gain.^20–22^ Such data are particularly sparse in adolescent populations, as their microbiome composition has largely been considered to be similar to that observed in adults.^17,23^ Moreover, aggregation of larger, longitudinal sample sets with high dimensionality and variable taxonomic abundances has presented substantial barriers in translating microbial data to meaningful health state insight. Therefore, in this exploratory study, we aimed to evaluate changes in body weight over the freshman year of college in relation to gut microbiome diversity, keystone taxa, and variability. In addition, we sought to explore potential drivers of latent community structure by constructing truncated fractional classification, considering our sizable and variable sample set. The study population was a cohort of incoming freshman college students living in residence halls on campus, grouped for microbiome analysis by weight gain, weight loss, and weight stable classification based on their longitudinal body weight change category. We hypothesized that the assessed microbial markers would distinctly track with each of the three weight change classifications. Findings provide insight into the complex relationship between changes in the gut microbiome community and weight in adolescents.

## Results

### Participant characteristics

**Figure 1.**
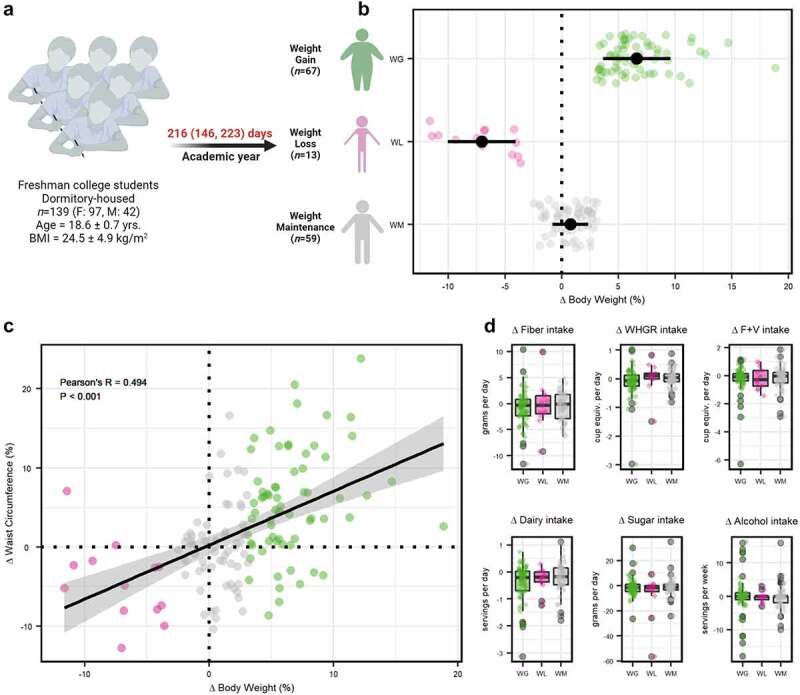
(a) Overview of study design. Participants that experienced weight gain (WG), weight loss (WL), or weight maintenance (WM) over the sampling period were assessed as discrete categories. (b) Change in body weight by weight change category. (c) Pearson’s partial correlation analysis between change in body weight and waist circumference. (d) Difference for change in fiber, whole grain (WHGR), fruit and vegetable (F+V), dairy, added sugar, or alcohol intake between WG, WL, and WM participants.

This study included 139 participants from a larger investigation that has been described previously.^24–26^ Briefly, this longitudinal study aimed to understand the impacts of social interactions, friends and friendship networks on nutrition, physical activity, and weight gain during the transition to college life. In the present study, we retained individuals who provided at least two stool samples (sample *n* = 372) at any time throughout the study duration to support this longitudinal analysis. Baseline participant characteristics are described in Table 1 and an overview of the study is displayed in Figure 1a. The median (IQR) number of days between the first and last fecal sample collection was 216 (146.5, 223) days. At baseline, a majority (*n* = 91, 65.5%) of participants were classified as having a body mass index (BMI) within the normal or underweight category (<25 kg/m^2^), though a substantial number of participants (*n* = 48, 34.5%) were classified as overweight or obese (BMI ≥25 kg/m^2^). Thus, to account for variability in initial body mass, we controlled for baseline BMI. Participants were distributed into three weight change clusters that included weight gain (WG; *n* = 67, 48.2%), weight loss (WL; *n* = 13, 9.4%), and weight maintenance (WM; *n* = 59, 42.4%). Specifically, WG, WL, and WM were defined as a gain of >3% of baseline weight, a loss of >3% body weight, and less than or equal to ± 3%, respectively. This classification was based on expert consensus recommending that weight maintenance be considered a weight change of ±3% of starting body weight, small weight fluctuations between ±3–5% weight change, and clinically relevant weight change at ≥ ± 5% change from baseline.^27^ Experts recommend that a percentage weight change is preferred over an absolute weight change as it better accounts for baseline body size.^27^

**Table 1.** Baseline demographic and anthropometric characteristics of study participants.

Characteristic	Total (*n* = 139)	WG (*n* = 67)	WL (*n* = 13)	WM (*n* = 59)
Age (years)	18.6 ± 0.7	18.6 ± 0.7	18.5 ± 0.3	18.6 ± 0.6
Sex, % (*n*)				
Male	30.2 (42)	29.9 (20)	38.5 (5)	28.8 (17)
Female	69.8 (97)	70.1 (47)	61.5 (8)	71.2 (42)
Race/ethnicity % (*n*)				
White	42.4 (59)	47.8 (32)	23.1 (3)	40.7 (24)
Black	12.9 (18)	13.4 (9)	7.7 (1)	13.6 (8)
Hispanic	25.9 (36)	25.4 (17)	23.1 (3)	27.1 (16)
Other	18.7 (26)	13.4 (9)	46.2 (6)	18.6 (11)
Height (cm)	167.1 ± 10.2	168.9 ± 10.2	168.5 ± 7.8	166.0 ± 10.9
Weight (kg)	68.9 ± 16.9	68.3 ± 16.5	71.8 ± 15.5	69.1 ± 17.9
Waist circumference (cm)	81.5 ± 13.1	80.2 ± 13.3	84.6 ± 12.7	82.2 ± 12.9
Waist/hip ratio	0.82 ± 0.07	0.81 ± 0.07	0.84 ± 0.08	0.83 ± 0.07
BMI (kg/m^2^)	24.5 ± 4.9	24.1 ± 5.1	25.1 ± 4.5	24.8 ± 4.8
BMI Categories (kg/m^2^), % (*n*)				
<18.5 kg/m^2^	4.3 (6)	4.5 (3)	7.7 (1)	3.4 (2)
18.5–24.9 kg/m^2^	61.2 (85)	64.2 (43)	46.2 (6)	61.0 (36)
25–29.9 kg/m^2^	23.7 (33)	20.9 (14)	30.8 (4)	25.4 (15)
≥30 kg/m^2^	10.8 (15)	10.4 (7)	15.4 (2)	10.2 (6)
BMI Dichotomized, % (*n*)				
Not overweight/obese	65.5 (91)	68.7 (46)	53.9 (7)	64.4 (38)
Overweight/obese	34.5 (48)	31.3 (21)	46.1 (6)	35.6 (21)

Data displayed as mean ± SD, unless stated otherwise. Abbreviations: WG, weight gain (gain of >3% of baseline weight); WL, weight loss (loss of >3% body weight); WM, weight maintenance (less than or equal to ±3% weight change).

No significant differences between weight change classification at baseline were detected for age, body weight, waist circumference, waist/hip ratio, or BMI (analysis of variance [ANOVA] tests, *P* ≥ .232); nor for self-reported lifestyle behaviors, including moderate to vigorous physical activity (MVPA), screen time, depression, sleep, and dietary factors (Kruskal–Wallis tests, *P* ≥ .090; Supplemental Table S1). In addition, the median number of days between the first and last sample collection was not significantly different by weight classification (Kruskal–Wallis test, *P* = .427). To better understand the relationships between anthropometric, dietary, and lifestyle factors at baseline, we conducted association analyses (total of 91 correlations). Of note, MVPA positively correlated with fruit and vegetable intake (Spearman’s *rho* = 0.466, *P.adj* = 7.69e-8), while weekly alcohol intake was negatively correlated with sleep (Spearman’s *rho* = −0.221, *P.adj* = 0.039) and positively correlated with added sugar intake (Spearman’s *rho* = 0.219, *P.adj* = 0.037). Further, red and processed meat intake was positively associated with added sugar intake (Spearman’s *rho* = 0.323, *P.adj* = 0.006). The mean daily consumption of dietary fiber for males (*n* = 42) and females (*n* = 97) was 18.69 ± 4.93 g/d and 14.73 ± 3.18 g/d, respectively, which fell below the Adequate Intake for both males (38 g/d) and females (25–26 g/d) per the Dietary Guidelines for Americans.^28^

Change in body weight (Δ post- vs. pre-body weight) was significant by weight classification (ANOVA, *P* = 0.006), with Bonferroni post-hoc comparisons showing significant distinction for all three categories (*P* ≤ .030; Figure 1b). Participants in the WG category gained an average of 6.6% over their initial body weight (+4.59 ± 2.61 kg: Supplemental Table S2), with the majority gaining over 5% (~70%, *n* = 45) and eight individuals gaining over 10%. In comparison, the WL group lost 7.1% of their initial body weight (−5.02 ± 2.34 kg), with three individuals losing more than 10% of their baseline body weight. Participants in the WM category maintained a relatively stable body weight, with an overall average weight change of ~0.8% (0.51 ± 1.16 kg). Relatedly, change in waist circumference and waist/hip ratio was significant by group (ANOVA tests, *P* < .001 & *P* = .010, respectfully). Bonferroni post-hoc comparisons revealed statistically significant waist circumference differences over the study period for WG participants (+5.44 ± 6.67%) compared to the participants in the WL and WM groups (−4.44 ± 5.06% & −0.01 ± 5.39%, respectively; *P* ≤ .022). A similar pattern was observed for waist/hip ratio (*P* ≤ .033) for participants with WG (+2.91 ± 6.03%), when compared to participants with WL (−1.04 ± 5.21%), and participants with WM (+0.26 ± 5.55%). Partial correlation analysis controlling for baseline BMI, sex, and individual participant revealed a significant positive association between overall change in body weight and waist circumference (Pearson’s *R* = 0.494, *P* = 9.468e-10; Figure 1c) and, to a lesser degree, overall change in body weight and waist/hip ratio (Pearson’s *R* = 0.203, *P* = 0.018).

Of note, there were no significant differences between the groups for change in depression (Kruskal-Wallis, *P* = .656) or moderate-to-vigorous physical activity (MVPA; ANOVA, *P* = .254), with participants maintaining relatively stable self-reported depression scores and physical activity relative to baseline values (Supplemental Table S3). Moreover, there were no significant differences between weight categories for change in nightly sleep hours (Kruskal-Wallis, *P* = 0.481). Finally, no statistically significant differences were detected for dietary factors over the study period between weight classifications (Kruskal–Wallis tests, *P* ≥ .209; Figure 1d; Supplemental Table S3).

### Longitudinal shift in microbial diversity displayed individualization for each weight change trajectory

**Figure 2.**
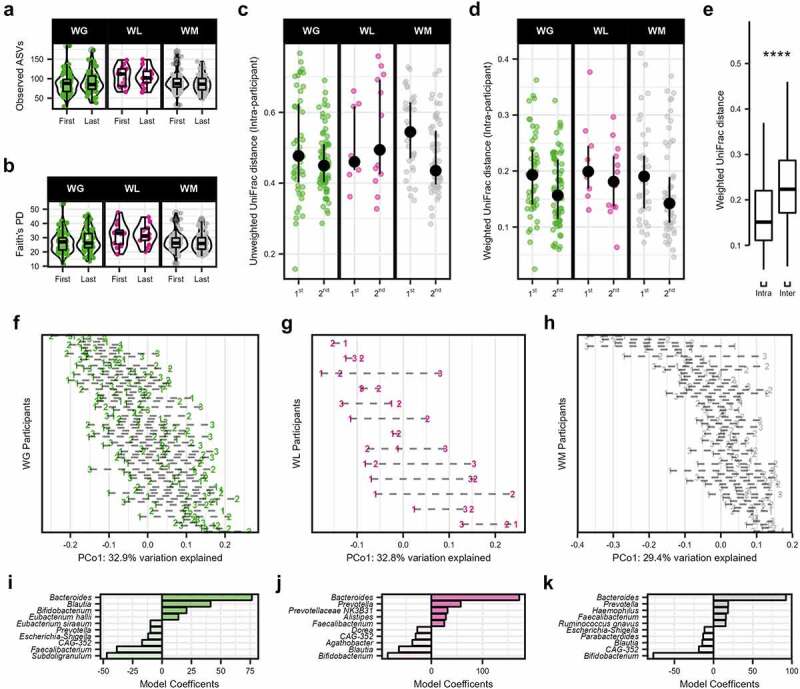
Gut microbiome diversity and community structure of freshman college students categorized by weight gain (WG), weight loss (WL), or weight maintenance (WM) change trajectories over the academic year. Alpha diversity, measured by (a) observed ASVs and (b) Faith’s PD. (c) Unweighted UniFrac and (d) weighted UniFrac distances within individuals (intra-individual) were calculated for the first (Δ first and second samples) and second (Δ first and third samples) distances. Median ±95% confidence intervals displayed in black. (e) Boxplots of the intra- and inter-individual weighted UniFrac distances for the 139 participants. Shifts in GM community at the genus level were observed across individuals on the first principal coordinate over the academic year of (f) WG participants, (g) WL participants, and (h) WM participants (weighted UniFrac). Each series of connected points represents a single individual in the study with the number representing the time point of the study in months. Model coefficients of the PERMANOVA analysis (model: Genus ∼ weight category × time) of weighted UniFrac distances for the (i) WG participants, (j) WL participants, and (k) WM participants displayed distinction and similarity. Genera with high coefficients (positive or negative) were visualized to distinguish the taxonomic drivers of the microbiome community over time between the three weight change trajectories. Only the top 10 genera are shown. In boxplots, the median is represented by the center line with the box representing the 1st and 3rd quartiles, whiskers extend 1.5× the interquartile range.

To evaluate whether weight change over the sampling period was associated with shifts in the gut microbiome, we first calculated observed amplicon sequence variants (ASVs) and Faith’s Phylogenetic Diversity (PD) of the 139 participants at their first and last fecal sample collection. Employing linear-mixed effect (LME) modeling, observed ASVs and Faith’s PD of microbial taxa varied significantly by group (LME, *P* ≤ 0.050); however, there was no significant difference between the three weight change categories after multiple test correction (*P.adj* ≥ 0.087; Figure 2a,b). There were also no significant interaction effects of time and weight classification for either alpha diversity metric (LME, *P* ≥ .169). To explore whether participant’s initial microbial diversity was associated with shifts in body weight, we constructed multiple regression models regressing change in body weight (post – pre-body weight) by baseline alpha diversity, controlling for BMI, sex, and participant. Change in body weight was not significantly associated with the observed ASVs or Faith’s PD (*R*^2^ ≤ 0.019, *P* ≥ .246), suggesting that initial alpha diversity was not a potential predictive factor for body weight change.

Despite the generally non-significant differences between weight classification groups by alpha diversity, unweighted and weighted UniFrac beta diversity metrics shifted significantly over time (accounting for all time points). Both distances were assessed to account for non-dominant (presence/absence: unweighted UniFac) and dominant (weighted by abundance: weighted UniFrac) microbes and displayed a low correlation to warrant inclusion of both metrics (Mantel test, *R* = 0.299, *P* < 2.2e-16; Supplemental Figure S1). Assessing unweighted and weighted UniFrac distances, permutational analysis of variance (PERMANOVA) revealed that most variation in community composition was explained by the individual participants (*R*^*2*^ ≥ 0.605, *P.adj* ≤ .002; Supplemental Tables S4 & S5). Composition also shifted significantly by weight change category over the duration of the study for weighted UniFrac, though this explained much less variation (interaction effect, *R*^2^ = 0.011, *P.adj* = 0.003) and was not significantly associated with unweighted UniFrac (interaction effect, *R*^2^ = 0.005, *P.adj* = .075). In addition, we calculated intra-participant distances between sequential stool collections and compared these between groups accounting for baseline BMI, sex, and the individual (as a random effect). Both unweighted and weighted UniFrac intra-participant distances decreased after the first pairwise comparison (LME, *P-adj* ≤1.2e-4) but did not differ by weight change category (*P.adj* ≥ .244; Figure 2c,d). Inter-individual distances were similarly not significant by group *(P.adj* ≥ .549; Supplemental Figure S2A-B), though were greater than intra-individual weighted UniFrac distances (+41.31% ± 22.53%, *P* < 2.2e-16) (Figure 2e), confirming that the gut microbiome is individualized as previously reported over a similar timeframe^29^. Indeed, individual effects within weight change categories accounted for greater variation over time for both distance matrices (PERMANOVA, *R*^*2*^ ≥ 0.257, *P.adj* ≤ .002). At the genus level, microbial shifts across individuals in all three weight change categories were also apparent by visualizing the time change of the first principal coordinates axis (Figure 2f,h; Supplemental Figure S3A-C). We examined the linear coefficients of PERMANOVA models using weighted UniFrac distances to assess taxonomic underpinnings of these shifts by weight change trajectories and dominant influences at the genus level. Sources of variation in WG community composition over time included positive contributions from notable taxa such as *Bacteroides*, *Blautia*, *Bifidobacterium*, and negative contributions from *Prevotella* and *Faecalibacterium* (Figure 2i). In contrast, *Prevotella* and *Faecalibacterium* were positive contributors and *Bifidobacterium* was a negative contributor for the WL and WM microbiomes, though, as with WG category, *Bacteroides* was dominant (Figures 2j,k).

### The ratio of Prevotella/Bacteroidetes did not significantly change across the WG, WL, and WM microbiomes

**Figure 3.**
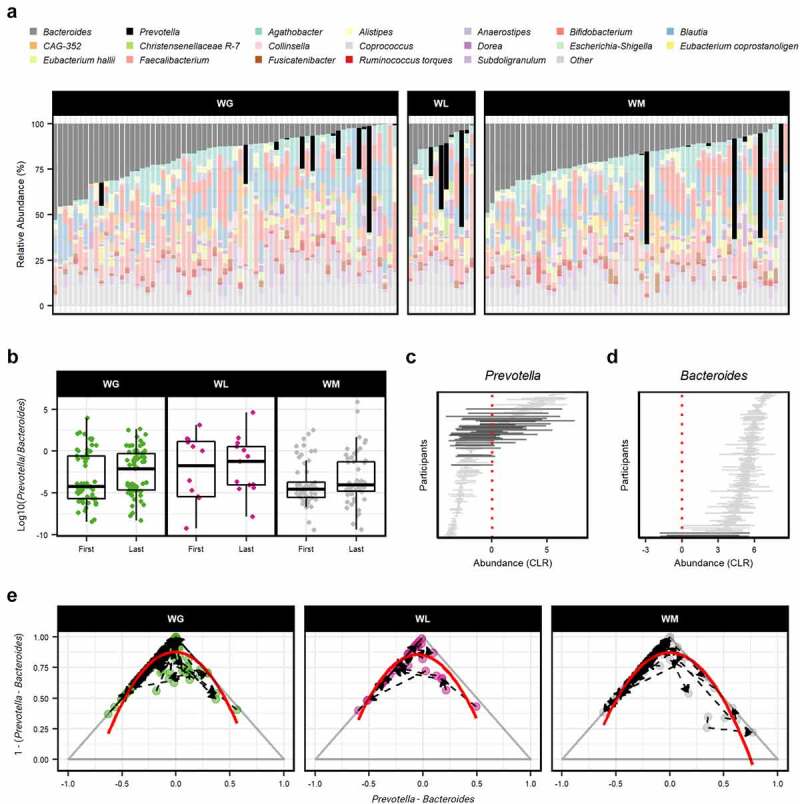
(a) The relative abundance (%) of bacterial genera in first-year college students’ gut microbiome samples. Each vertical bar represents a separate stool sample taken at the beginning of the study period. Samples are categorized by weight gain trajectory over the academic year (weight gain [WG], weight loss [WL], and weight maintenance [WM]) and are ordered according to the abundance of *Bacteroides*. Samples from 139 individuals in total: WG = 67, WL = 13, and WM = 59, respectively. The top 20 genera are displayed with the remaining taxa collapsed into the category “other”. (b) Log10 ratio of *Prevotella*-to-*Bacteroides* displayed a significant effect of weight change category (linear-mixed effects model, *P* = .033). In boxplots, the median is represented by the center line with the box representing the 1st and 3rd quartiles, whiskers extend 1.5× the interquartile range with outliers individually plotted. Temporal variation of (c) *Prevotella* and (d) *Bacteroides* represented by horizontal lines (CLR abundance range) over the study duration for each participant. Bold lines indicate potential state-shift by intersecting the estimated tipping point (red dashed line). (e) Ternary plots for WG, WL, and WM participants depicting the relationship between *Prevotella* (dominate at the right vertex), *Bacteroides* (dominate at the left vertex), and the other genera (other = 1 – (*Prevotella* – *Bacteroides*); dominate at the top vertex). Curvature of transitions within and between *Prevotella*- and *Bacteroides*-dominated regions is denoted by a polynomial regression line (red) which accounted for the majority of explained variance (WG = 81.8%, WL = 88.5%, WM = 89.9%).

Consistent with the genera that contributed to variation in beta diversity over time, stool samples from participants at baseline were dominated by *Bacteroides* (mean relative abundance: WG, 16.9% ± 13.5% SD; WL, 13.9% ± 15.7%; WM, 18.2% ± 10.8%), *Blautia* (WG, 11.9% ± 7.6%; WL, 9.2% ± 6.3%; WM, 11.8% ± 7.5%), *Faecalibacterium* (WG, 10.4% ± 9.4%; WL, 12.5% ± 11.6%; WM, 7.3% ± 6.0%), and *Bifidobacterium* (WG, 9.0% ± 9.1%; WL, 7.9% ± 8.6%; WM, 10.3% ± 9.8%; Figure 3a). Notably, *Prevotella* was much more disparate between groups with participants, with WL displaying a greater abundance (WG, 2.6% ± 8.7%; WL, 10.2% ± 17.3%; WM, 3.6% ± 13.1%), consistent with the driving taxa in beta diversity shifts in this weight classification. The broad coverage of *Bacteroides* and sparse (though pronounced, where occupant) abundance of *Prevotella* was apparent and selected for further analysis due to relevance of these two genera in weight change^30^ and long-term diet.^31^ Of the participant’s first stool collection, *Bacteroides* displayed a left-skewed abundance pattern and of all genera had the lowest evidence of multimodality via prediction strength index (PSI) (0.23). Conversely, *Prevotella* had moderate support for bimodality (PSI = 0.71), supporting that this microbe was much less abundant in the gut microbiome across individuals. Calculating the ratio between these taxa (see Materials and Methods), we did not observe significant effects for time or interaction of time and weight change category (LME, *P* ≥ .131; Figure 3b). However, there was a significant effect of weight change category (LME, *P* = 0.033), with multiple comparison testing revealing a nearly significant difference between participants with WL and WM (*P.adj* = 0.050).

To better understand the stability of these genera and whether they shifted to an alternative state over the sampling period, we performed calculations of multimodality based on bootstrapped potential analyses, as previously described.^32^ Referred to as the ‘tipping point’ (TP), this analysis estimates the intermediate unstable region where such shifts may occur. Participants that intersected the calculated threshold for *Prevotella* (TP = 0.013) and *Bacteroides* (TP = 0.002) displayed significant increased temporal deviation compared to participants that did not (Wilcoxon rank sum test, *P* = 2.665e-15 and *P* = 0.003, respectfully; Figure 3c,d). These results are suggestive of an avoidance of temporal stability at the intermediate abundance range (low vs. high) and a preference for either a *Bacteroides*- or *Prevotella*-dominated state. This was also apparent by the negative correlation between these two genera accounting for all samples (Spearman’s *rho* = −0.213, *P* = 3.5e-5).

Adapting a longitudinal and continuous analytical approach inspired by Levy and colleagues,^33^ we examined the potential for individuals in the three weight change categories to shift between community types (i.e., *Bacteroides*- vs *Prevotella*-dominated). Representing potential codominance between *Prevotella* and *Bacteroides*, the intermediate space in the middle of each ternary plot was generally empty (Figure 3e). Visually, there was an apparent curvature of individual trajectories that shifted along a *Prevotella*- or *Bacteroides*-dominated gut microbiome, with few transitions between these states. Polynomial regression models constructed for participants with WG, WL, and WM all accurately mapped to this curvature, accounting for ≥81.8% of the total variance.

### Intra- and inter-participant taxonomic variation between weight change trajectories

**Figure 4.**
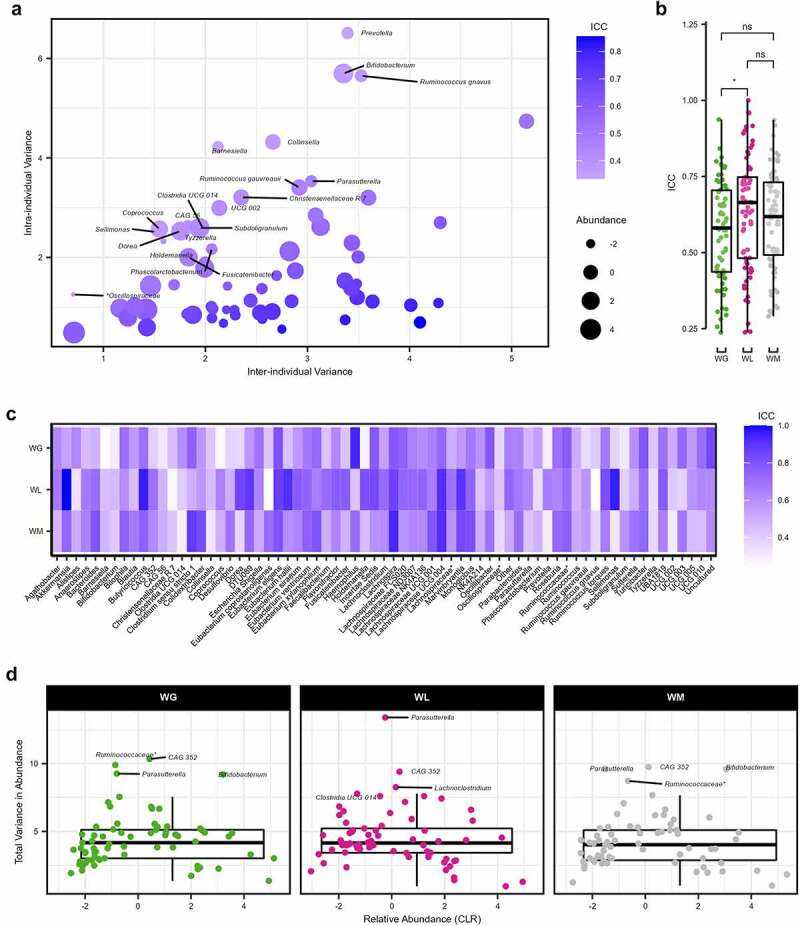
Variation of the gut microbiome at the genus level. (a) Relationship between the intra-individual and inter-individual variations of genera for all samples. Dots colored by intraclass correlation coefficients (ICC) and sized according to the center log-ratio (CLR) relative abundance. Genera with an ICC ≤ 0.50 are labeled by name. (b) Boxplots of the ICC for each genus within weight gain (WG), weight loss (WL), or weight maintenance (WM) participants (Wilcoxon rank sum test, *P = .023). (c) Heatmap of the ICC for each genus by weight change trajectory. (d) Total variance (intra-individual + inter-individual variance) of each genus for WG, WL, and WM participants, plotted against the CLR relative abundance. Outliers for each group are labeled by name. For boxplots, the median is represented by the center line with the box representing the 1st and 3rd quartiles, whiskers extend 1.5× the interquartile range. Features with an asterisk are listed at the family as the genus was unclassified.

To assess the intra- and inter-participant microbial abundance variation, we aggregated ASV reads to the genus level for samples from all time points (*n* = 372) after filtering out rare taxa and performing a center log-ratio (CLR) transformation on relative abundance values. As described in the Methods section, the resultant 69 features were then passed independently through linear-mixed effect models (LMM) for all samples and then by WG, WL, and WM status with participant as a random variable. As described recently by Olsson and colleagues (2022), intraclass correlation coefficients (ICC) were calculated from the intra- and inter-individual variances extracted from each LMM.^29^ The median ICC of all samples was 0.59 (IQR = 0.24), demonstrating the variation between individuals was greater than the variation within individuals. However, 20 taxa had an ICC ≤ 0.50, indicating greater variation within participants (Figure 4a). Notable features with the lowest ICC included *Prevotella*, *Bifidobacterium*, *Ruminococcus gnavus*, *Collinsella*, and *Barnesiella* (ICC ≤ 0.381; Supplemental Table S6). While many of these taxa displayed a non-trivial relative abundance (i.e., ≥1%), there was no significant correlation between CLR relative abundance and ICC (Spearman’s *rho* = −0.122, *P* = .316). Comparing the ICC between the three weight change categories, participants with WL had a significantly greater overall value compared to participants with WG (Wilcoxon rank sum test, *P*= .023; Figure 4b), suggestive of increased taxonomic stability within individuals while displaying greater distinction between individuals. No significant differences were detected comparing participants with WG to WM or WL to WM (*P* ≥ .230). Examining the ICC for each genus of participants with WL, notably high values (ICC ≥ 0.80) were apparent for *Akkermansia*, *Butyricicoccus*, *Dorea*, *DTU089*, *Eubacterium eligens group*, *Eubacterium hallii group*, *Holdemanella*, *Incertae Sedis*, *Lachnospira*, *Lachnospiraceae UCG 004, Marvinbryantia, Ruminococcus torques group*, and *Sellimonas* (Figure 4c; Supplemental Table S7). In comparison, participants with WG had lower values (ICC ≤ 0.50) for notable features such as *Anaerostipes*, *Bacteroides*, *Faecalibacterium*, *Prevotella*, and *Ruminococcus gauvreauii group* compared to participants with WL and WM indicating greater within-participant variability. The total variance in abundance was not significantly correlated with genus CLR relative abundance (Spearman’s *rho* = 0.124, *P* = 0.308). Moreover, there was no significant difference in total variance between weight change categories (Wilcoxon rank sum test, *P* ≥ .330). Of note, the genera that had the greatest variation (i.e., the least stable) were similar between weight trajectories. These included the outliers (+1.5 IQR) *Parasutterella* and *CAG 352* shared across all three weight categories, and an unclassified Ruminococcaceae genus and *Bifidobacterium* for participants with WG and WM (Figure 4d).

### Latent community types associate with weight change trajectories and lifestyle factors

**Figure 5.**
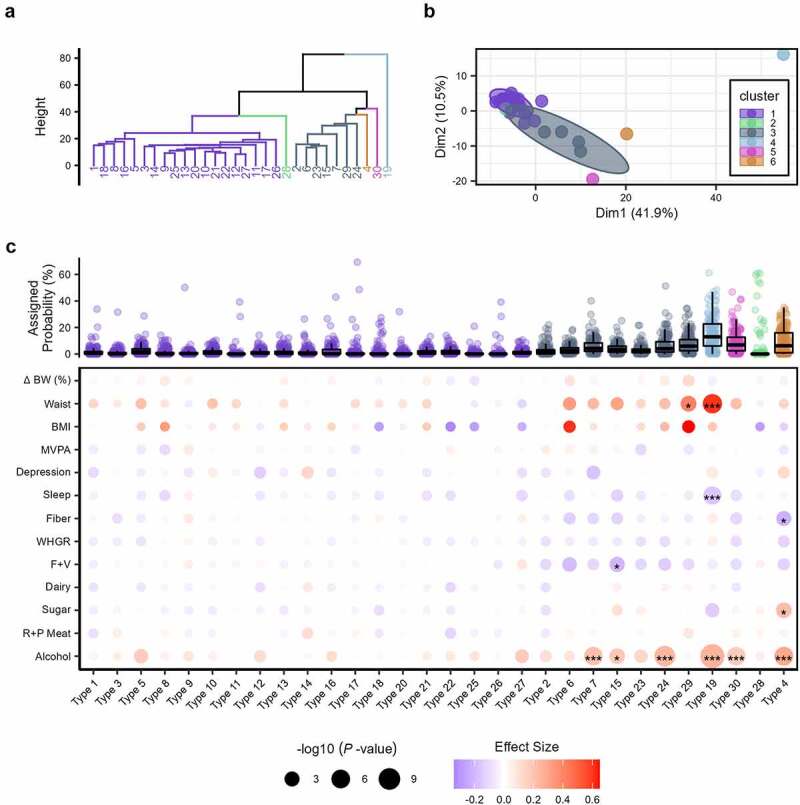
(a) Dendrogram of the six hierarchical clusters of community types from participants’ baseline sample. (b) Plot of the six hierarchical clusters of community types from participant’s baseline sample. Each cluster is denoted by a different color and the numbered nodes represent the latent community types. Ellipses for clusters 1 and 3 are 95% confidence intervals. The composition of each of the 30 community types are displayed in Supplemental Figure S4. Ward’s minimum variance method was used for hierarchical clustering. (c) Boxplots of the assigned probability per microbiota community type are organized and colored per the six clusters at the top. Below a dot plot illustrates the associations derived from Dirichlet regression models between the 30 latent community types and anthropometric, behavioral, and dietary factors. Each circle represents a separate association with the size indicating the significance (-log10 (*P*-values)) and the color the effect size (hue) with its direction (red: positive; blue: negative). Asterisks within a circle denote a significance after adjustment with the Benjamini–Hochberg method, *P.Adj < .05, ** *P.Adj* < .01, *** *P.Adj* < .001. Abbreviations: BW, body weight; BMI, body mass index; F+V, fruit and vegetable intake; R+P meat, red and processed meat.

We derived the community-type likelihood of all individual samples at the genus level utilizing latent Dirichlet allocation (LDA), an unsupervised machine learning approach that allows fractional membership based on the probability of assignment across community types (as opposed to exclusive assignment).^34^ First, to assess the predictive potential of community type in weight change, we specified 30 latent community types based on model fit for 160 genera (comprised of 1,169 ASVs) (Supplemental Figure S4). Hierarchical cluster analysis revealed six groupings (Figure 5a), with apparent separation for community types dominated by *Bacteroidetes* (Cluster 4; Type 19), *Faecalibacterium* (Cluster 5; Type 30), *Bifidobacterium* (Cluster 6; Type 4), and, to a lesser extent, *Prevotella* (Cluster 2; Type 28) (Figure 5b). Clusters 1 and 3 contained 19 and 7 community types, respectfully, and displayed a greater degree of shared taxa contributions for each type. For example, the top five genera for community type 18 within cluster 1, were *Alistipes*, *Bacteroides*, *Clostridia UCG 014*, *Eubacterium siraeu*, and *Faecalibacterium*. Assignment probability for each of these taxa was within the range of 10–20%. Similar community types with more varied taxa contributions included types 2, 8, 10, 11, 14, 15, 17, 20, 21, 22, 25, and 26.

The per-sample-per-community type probabilities for the first microbiome samples were extracted for each weight change classification controlling for sex and baseline BMI using Dirichlet regression models. This statistical approach was indicated as we assessed multiple predictive variables with outcome variables as the 30 compositional community types (i.e., values across the subgroups add up to 1 for each observation). Comparing per-community-feature-probabilities across weight change categories at baseline, type 12 (dominated by *Oscillospiraceae UCG-002*), 22 (dominated by *Lachnospiraceae NK4A13*), 28 (dominated by *Prevotella*), and 30 (dominated by *Faecalibacterium*) had a greater likelihood of occurrence in participants with WL, whereas type 19 (dominated by *Bacteroides*) had a greater likelihood of occurrence in participants with WG and WM (*P.adj* ≤0.041; Supplemental Figure S5). We next considered an unbiased assessment, disregarding weight trajectory assignment (e.g., WG, WL, and WM), of the samples collected after baseline might demonstrate unappreciated community patterns. Using GM samples from the second and third longitudinal collection, we assessed anthropometric (i.e., change in percent body weight, waist circumference, and BMI), behavioral (MVPA, depression, and sleep), and dietary (i.e., intake of fiber, whole-grains, fruit and vegetables, dairy, added sugar, red and processed meat, and alcohol) factors with multivariate-adjusted Dirichlet regression models, controlled for sex and baseline BMI. After multiple testing correction, 12 significant associations with microbial community type were detected (Figure 5c). Alcohol intake (number of weekly drinks) was the most frequent, statistically significant correlate to community type (i.e., six significant associations) and, to a lesser extent, waist, sleep, and added sugar and fruit and vegetable intake (i.e., ≤2 significant associations; *P.adj* ≤ .027; Table 2). Waist circumference displayed the greatest effect size (beta-coefficient), with positive values from community types 19 (dominated by *Bacteroides*) and 29 (dominated by *Blautia*). In addition, sleep was negatively correlated with type 19 (*P.Adj* = 1.81e-04). Notably, community type 4 (dominated by *Bifidobacterium*) was positively associated with alcohol and sugar intake (*P.Adj* ≤.019) and negatively associated with fiber intake (*P.Adj* = .035).

**Table 2.** Significant community-type associations with anthropometric, behavioral, and dietary factors of all participant samples taken after the baseline collection.

Factor	Community	Effect Size	*P*-Value	*P.Adj*
Alcohol	Type 19	0.256	1.16e-12	4.54e-10
Alcohol	Type 24	0.244	8.79e-09	1.71e-06
Waist Circumference	Type 19	0.602	2.86e-07	3.71e-05
Alcohol	Type 4	0.291	8.96e-07	8.74e-05
Alcohol	Type 7	0.213	1.57e-06	1.22e-04
Alcohol	Type 30	0.178	1.91e-06	1.24e-04
Sleep	Type 19	−0.159	3.25e-06	1.81e-04
Waist Circumference	Type 29	0.410	3.29e-04	.016
Added Sugar	Type 4	0.223	4.76e-04	.019
Alcohol	Type 15	0.191	4.56e-04	.019
F+V	Type 15	−0.208	7.85e-04	.028
Fiber	Type 4	−0.217	.001	.035

Data ordered and displayed by *P.Adj* value (descending). Effect sizes are beta-coefficients derived from multivariate-adjusted Dirichlet regression models, controlled for sex and baseline BMI. Abbreviations: F+V, fruit and vegetable intake; *P.Adj*, adjusted *P*-value using the Benjamini-Hochberg procedure.

## Discussion

To our knowledge, this is the first longitudinal study to observe a statistically significant relationship between weight change and the structure of the gut microbiome in dormitory-dwelling emerging adults during the first year of college. The average weight change of participants was +2.6% (+1.8 kg), with almost half of the participants gaining weight over the course of their first year. As a proxy for body composition, weight gain was significantly associated with waist circumference, indicating a likely increase in body fat.^35^ Notably, the type of weight change was associated with different structural shifts within the community of gut microbes and displayed distinct variability in select taxa in our adolescent population. However, in contrast to our original hypothesis, we did not observe an overall distinct tracking of gut microbiome diversity, keystone taxa, and variability in latent structure with each of the three weight change classifications.

As reported previously by Olsson et al. (2022), we observed greater overall inter-individual variation in dissimilarity and taxonomic composition in the gut microbiome.^29^ In agreement with Olsson and colleagues, we also noted that differences within individuals over time can be quite variable and may pose a significant source of confounding when examining health outcomes. However, in comparison to their older population (50–65 years) living in a stable situation, our participants underwent a disruptive life change transitioning into independent living on a college campus. Core genera detected in their study such as *Prevotella* and *Bifidobacterium* displayed a much greater ICC in comparison to our results. This discrepancy may have been due to the environmental pressures and significant changes in anthropometric outcomes we noted in our sample. Moreover, *Prevotella* was relatively sparse in our samples which may have skewed the variation we observed in relation to other taxa. Others, such as the most prevalent genus, *Bacteroides*, displayed a greater degree of stability. In addition, our results align with the high between-person differences for abundance of *Akkermansia* from both longitudinal and cross-sectional research.^29,36^ Such inter-person variability in select microbes like *Prevotella*, *Bacteroides*, and *Akkermansia* are important considerations in the context of body weight control and, when accounted for in an intervention setting, may improve the detection of significant outcome changes.^30,37–39^

Interestingly, weight change was not significantly related to microbial richness or phylogenetic diversity (alpha diversity) between weight change categories. Moreover, baseline alpha diversity was not predictive of weight change. Together, these findings demonstrate that alpha diversity did not have a significant influence on body weight change over the course of the study. Few clinical studies have evaluated changes in microbial diversity in relation to weight change over time which makes comparison to other studies difficult, especially in adolescent or youth populations. Recently, a meta-analysis assessing microbiome-related metrics reported increased alpha diversity levels, pooling 47 trials with 1,916 participants.^40^ However, included studies investigated deliberate weight loss regimens and procedures (energy-restricted diets, pharmacotherapy, bariatric surgery) in middle-aged populations that are likely not representative of the nutritional and behavioral stress experienced by college students. More relatedly, small cross-sectional studies (*n* = 53) have observed differences in richness and evenness between children with BMI percentiles between the 15^th^ and 94^th^ percentiles.^41^ Conversely, a large cohort study of 295 school-aged children (KOALA Cohort Study) found no differences in microbial diversity and richness when comparing children of different weight status 6–7 years after birth.^42^ Authors of the KOALA Cohort Study suggest that they did not observe a relationship because they were able to control for more confounding factors (e.g., diet, lifestyle factors) than most studies. While we report similar covariate control and findings, the stability of alpha diversity is noteworthy considering our longitudinal assessment during a period of expected weight gain and major lifestyle changes such as the first year of college.

In comparison, the microbial community significantly shifted, showing a high degree of variation between and within individuals. This shift was more pronounced between the first and second collections (first distance), with dissimilarity decreasing comparing intra-individual differences of the first and third collections (second distance). These results perhaps indicate a shift closer to baseline composition, considering the initial environmental changes experienced by the participants. Future research may explore changes in microbial communities with more frequent sample collections as well as continuing throughout the advancement of college (i.e., sophomore year, independent living off campus, etc.). While community shifts were apparent for all three weight change categories, the genera that drove the changes in participants with WG compared to WL and WM microbial structure over the academic year displayed some distinction. Protracting elements of the WG shift included *Bacteroides*, *Blautia*, *Bifidobacterium*, while detractors were *Prevotella* and *Faecalibacterium*. In the case of many of these taxa, we observed the opposite for the microbiome of participants with WM and WL, with positive contributions from *Prevotella* and *Faecalibacterium* and negative contributions from *Bifidobacterium*. Regardless of weight change trajectory, we noted high coverage of *Bacteroides* in samples. In comparison, the occurrence of *Prevotella* was much less, but when it was present in a sample, its relative abundance was pronounced. These results align with previous reports from larger adult cohorts,^33,43,44^ where there was a clear ecological dichotomy in the presence of these genera. *Prevotella*-dominated microbiomes were rare in our overall sample, though did appear to be associated with weight change trajectory assessing the *Prevotella*/*Bacteroides* (P/B) ratio. This ratio has been previously shown to predict weight loss,^30,37^ and is associated with a dietary intake rich in carbohydrates, resistant starch, and fibers, whereas a diet high in fats but low in fibers is associated with a low P/B ratio.^45^ In the present study, we noted a significant, though modestly greater P/B ratio in the WL vs. WM microbiome. Interestingly, all participants reported a low consumption of fiber, and we did not detect significant differences in fiber, whole grain, or fruit and vegetable intake between participants with WG, WL, and WM. This may have been related to our dietary data collection tools and future work in this population should use more precise assessment methods such as 24-hour dietary recalls collected in multiple passes by nutrition professionals.

Our results reinforce that the P/B ratio can be resistant to change,^30,33,46^ particularly considering our participants were free-living and not enrolled in a controlled dietary intervention. Such resistance has been speculated to be due to a barrier greatly limiting the transition between *Prevotella*- and *Bacteroides*-dominated gut communities.^33^ Assessment of longitudinal shifts in alternative states of *Prevotella* and *Bacteroides* revealed an intermediate unstable region where, once crossed, resulted in a significant change in abundance to an alternative, presumably more stable, state. Shifts were much more apparent with the genus *Prevotella*, although did occur with *Bacteroides* when change in abundance crossed a calculated threshold (i.e., the tipping point). The bimodality of *Prevotella* is in agreement with previous reports including longitudinal assessments,^32,47^ and our data support that both *Prevotella*- and *Bacteroides*-dominated microbial communities shift more along an apparent gradient,^48^ and are highly individualized. Moreover, there was a noticeable barrier for transition between compositional states. This aligns with the anti-correlative relationship between *Prevotella* and *Bacteroides* in human microbiome and the resistance to simple transition to alternative stable states. Levy and colleagues postulated that this is based on several host factors and microbiome dynamics that create regions impermissible (resistant) to transit through microbiome state-space.^33^ Indeed, we noted this same curvilinear trend in each individual microbiome trajectory after mapping polynomial regression models for the three weight change categories. As hypothesized, to transition to either a *Prevotella*- or *Bacteroides*-dominated state, an individual’s gut microbiome must deplete both genera and pass through a permissive region in state space.^33^ This has important implications for engineering the microbiota in accordance with improving host health, including when to make such attempts in an individual’s life cycle, how to modify diet, and when/where targeted antimicrobial agents, fecal microbiota transplantations, probiotics, and other therapeutics may be best leveraged.

Observational studies such as ours and others report that direct transitions from a *Bacteroides*- to a *Prevotella*-dominated state (and vice versa) are more infrequent.^33^ Unsurprisingly, spurring this transition is of great therapeutic interest in relation to multiple health outcomes as these genera have been identified as important correlates with diet and aging.^44^ More recently, a randomized control trial in obese adolescents (14–18 years old) found that fecal microbiota transplantation from lean donors that had a high P/B ratio was better able to engraft in the recipient’s gut, which promoted shifts in community composition.^49^ Specifically, from *Bacteroides* to *Prevotella* dominance, showcasing the ability of *Prevotella* taxa to outcompete *Bacteroides* taxa in ecological dominance. Depending on several host and environmental factors, this may be due to higher bacterial growth rates of *Prevotella*, depending on internal and external conditions.^50^ Interestingly, this pattern of unbalanced occurrence has been observed across multiple body sites in humans, including the airways, gut, skin, oral cavity, and vagina.^51^ This occurrence pattern is interesting considering that these genera are phylogenetically related (i.e., from the order Bacteroidales), yet display markedly different functionalities. For example, *Prevotella* has a higher fiber utilizing capacity and total short-chain fatty acid production.^52^ In the context of body weight shifts, a microbiome with a greater P/B ratio appears to support weight decrease when supplied with certain nutritional inputs, such as a high fiber/whole-grain diet.^30,53,54^ We did not observe changes in nutritional factors in WG, WL, and WM participants that would promote such a change, including increased dietary fiber, whole-grain, and/or fruit and vegetable intake. This potential lack of priming the functional capacity of a high P/B microbiome may have been partially responsible for some of the observed weight change and could have important implications for diet modification in these individuals.

While we conducted several analyses exploring *Bacteroides*, *Prevotella*, and other keystone taxa, this approach may lend oversimplification to the immense dimensionality of microbiome data, especially considering the relatively large number of samples and ASVs captured in our analysis. This was our motivation for dimensionality reduction via the formation of latent communities grouped by co-occurring bacterial taxa and, in addition to, hierarchical clustering. Rather than form discrete memberships, a notion forwarded as ‘enterotypes’,^43^ we sought to leverage LDA modeling for its fractional membership. This tool has been implemented with cross-sectional designs and has shown recent promise for interpreting latent structures within the human gut microbiome.^55,56^ The more pronounced community types identified in our analysis, by abundance and hierarchical separation, were dominated by several genera we had previously identified in our PERMANOVA models and relative abundance assessment, such as *Bacteroides*, *Prevotella*, *Blautia*, *Bifidobacterium*, and *Faecalibacterium*. The community types that are significantly associated with the microbiome of WL participants, including types 12, 22, 28, and 30, suggest these bacterial communities may play a role in promoting weight loss. Considering the vulnerability of our population, deleterious lifestyle factors (i.e., mood disturbance, dietary intake, alcohol consumption, etc.), and within-healthy range starting BMI, this should not be viewed as a necessarily beneficial outcome. Even so, by percent assigned probability, types 28 and 30 were notable features and were all but dominated by *Prevotella* (with minor contributions from an unclassified genus from the Lachnospiraceae family, *Anaerostipes*, *Coprococcus*, and *Sutterella*) and *Faecalibacterium* (with minor contributions from an unclassified genus from the Lachnospiraceae family, *Blautia*, *Coprococcus*, and *Fusicatenibacter*), respectfully. While not the intent of this community analysis, the feature importance of these genera in WL participants is notable as they have been considered commensal fixtures within a healthy gut microbiome. For example, species within the *Faecalibacterium* genus, such as *F. prausnitzii*, are known to have anti-inflammatory properties critical to colonocyte health^57^ and are significant butyrate producers.^58^ Moreover, both *Faecalibacterium* and *Prevotella* have been associated with healthier dietary patterns in Western populations (i.e., lower fat, greater intake of whole grains and fiber, increased diet quality, etc.),^54,59,60^ with increased abundance of *Prevotella* linked to weight loss success.^30,37^ Conversely, community type 19, almost completely dominated by *Bacteroides* (with minor contributions from an unclassified genus from the Lachnospiraceae family, *Flavonifractor*, *Ruminococcus torque*, and *UBA 1819*), was more associated with the microbiome of WM and WG participants. Depending on the dietary input, *Bacteroides* has been shown to be positively associated with weight gain^61^ and a determinant of fat loss success.^30^ It should be noted that the genera of *Faecalibacterium*, *Bacteroides*, and *Prevotella* all have diverse species, and this important nuance was likely not captured as we were limited in our taxonomic resolution. Indeed, of the 1,000+ ASVs after filtering, we had condensed to 160 genera prior to community modeling. Regardless, the associations found for the three weight loss trajectories are consistent with patterns at the genus level noted in the context of body weight and composition regulation.

As has been reported in previous work employing LDA modeling^55,56^, there is utility in providing a more truncated fractional classification to the highly complex community of microbes in the gut, especially when assessing larger sample sizes. Our approach sought to afford more meaningful insight into the overall structure of the gut microbiome over time. Longitudinal assessments are critical as capturing shifts in health states with tools like LDA may yield diagnostic value and provision for identification of candidate communities and taxa as therapeutic targets. Importantly, LDA learns patterns of co-occurrence of features rather than clustering observations based on distance measures. The derived community types may cluster based on factors explored here, such as environmental exposure and associated factors moderated by behavior (i.e., stress, habitual diet, etc.). Indeed, upon exploration of the associations between community types and anthropometric, dietary, and behavioral factors, we noted several important trends relating to body weight and composition modulation. For instance, waist circumference had significant correlations with several of the latent community types, while change in body weight and BMI did not. As a proxy for central adiposity, these findings suggest the significance of increased body fat in this region. Community Type 19, dominated by *Bacteroides*, and 29 dominated by *Blautia* (with minor contributions from an unclassified genus from the Ruminococcaceae family, *Lachnoclostridium*, *Monoglobus*, and *Turicibacter*). In rodent models, high-fat diets and bile acid influx in the gut have been associated with *Blautia*. ^62^ In humans, *Blautia* has been associated with visceral fat in a large sample of twins from the TwinsUK cohort (*n* = 3,666).^63,64^ Notably, *Blautia* in our analysis was present in many community types (Types: 2, 3, 6, 8, 9, 11, 13, 15, 21, 23, 25, 26, and 30) with its near exclusive assignment in only one community Type (i.e., type 29). Thus, these results provide support for fractional assignment of LDA to identify the pervasiveness and ability of taxa like *Blautia* to co-operate/habitate with other microbes, whether due to ecological pressures and requirements, mutually beneficial relationships, function, etc. Indeed, the high frequency of co-occurrence across community types is plausible considering *Blautia* contains many stress-related genes, can utilize many different sugars, and produces organic acids (chiefly acetic acid as well as some long-chain fatty acids) and an array of secondary metabolites.^65^ Many of these secondary metabolites, like non-ribosomal peptides, polyketides, lanthipeptides, and bacteriocins, may function to aid nutrient acquisition, chemical communication, and inhibit/promote colonization of other microbes,^65–67^ which reflects in its ability to reside in a diverse range of hosts.^68^

Other significant associations were unsurprising considering the exposures that are present in a college setting, such as increased ingestion of alcohol and sleep disturbance. Of the 12 significant associations observed in the current study, half of those between community types and anthropometric, dietary, and behavioral factors were related to alcohol intake. Excessive alcohol use has been suggested to have a profound effect on the gut microbiome by depleting several health associated taxa, such as *Akkermansia*, *Faecalibacterium*, *Prevotella*, *Ruminococcus*, and increases in potentially deleterious microbes, such as Enterobacteriaceae.^69^ Of note, community type 4, which was largely driven by *Bifidobacterium*, was positively associated with alcohol and sugar intake. Several *Bifidobacterium* strains have been identified for their high capacity for ethanol metabolism (encoding the enzymes alcohol dehydrogenase and acetaldehyde dehydrogenase) and potential as a probiotic agent for alcohol detoxification in humans.^70–72^ However, recent work in mice suggests that ethanol does not significantly contribute to changes in the gut microbiota, rather the short-chain fatty acid acetate that enters the gut after being produced by the liver.^73^ Regardless, *Bifidobacterium* has been found to be positively associated with sucrose intake in patients with type 2 diabetes.^74^ Various strains of Bifidobacterium have long been known to ferment various sugars, including Glucose, Lactose, Galactose, Mannitol, and Xylose.^75^ Moreover, *Bifidobacterium* possesses a bifid shunt, which could efficiently produce adenosine triphosphate from glucose.^76^

To the best of our knowledge, no longitudinal studies similar to ours in scale and duration have been completed in emerging adult populations. Many existing studies on gut microbiome structure in relation to weight have focused on adult populations. Overall, these studies among adults have demonstrated that community structure and species richness may be associated with obesity or weight gain over time.^77^ However, research indicates significant differences between the adult and adolescent microbiome, and it has been suggested that abundance may drive these differences in community composition.^17,78^ Previous studies have also reported that weight is negatively associated with microbial diversity, but few studies have looked at this relationship longitudinally, especially in a vulnerable adolescent population at risk for excessive weight gain. These gaps in the current literature highlight two major strengths of this study: the adolescent population and the longitudinal study design. In particular, a free-living adolescent sample during a time of expected weight gain adds real-world relevance for understanding the interplay between weight change and gut microbiome composition. Additional strengths of this study include the larger sample size and the racial and ethnic diversities of the sample, enhancing the generalizability of the findings. Limitations of this study include unbalanced samples across the weight change groups as a result of a convenience sample, and high attrition rates for longitudinal fecal sample collection. We assessed change in individual BMIs, an important indicator of health trajectories and often shown to be linked to health behaviors. Several of the analyses presented were limited to categorical classifications of BMI change which may have limited the clinical impact of this work when compared to continuous data approaches. Further, we want to acknowledge limitations in assessing BMI, particularly among a diverse population, and emphasize that in 2023, the American Medical Association indicated that BMI should no longer be used alone as a diagnostic tool for obesity.^79,80^ Regardless, this study serves as an important first step in uncovering the many underpinning elements of the relationship between the gut microbiome, lifestyle factors, and weight gain in college students. Further studies assessing the impact of the gut microbiome in mediating the relationship between lifestyle factors and weight gain may help us understand the phenomenon of college weight gain. This understanding may better inform prevention efforts and minimize weight gain throughout early adulthood. Future research should focus on more frequent sampling, incorporation of additional relevant sample types (i.e., fecal and plasma metabolomics), and tighter study control. Researchers may also sample both adults and adolescents in future longitudinal studies analyzing changes in the gut microbiome in relation to changes in weight to compare differences by life stage. A focus on energy balance studies may also be beneficial for elucidating the role of microbes in weight change trajectories.

In conclusion, this longitudinal study highlights the importance of individual variability, drivers of shifts in community structure, and the relationship with nutritional and behavioral factors on the gut microbiome of adolescents of three weight change categories in a college setting. While we show many qualities of an adult-associated assemblage of gut microbes, such as apparent gradients between shifts in taxonomic dominance, we also highlight differential variability. In our well-phenotyped cohort, we note that tracking, let alone, predicting weight change by any of the numerous accepted determinants (i.e., diet, microbiome, starting weights, psychological readouts, etc.) is extremely difficult. While we did note some differential shifts in taxa associated with weight change classification, such results will require reinforcement via replication in a distinct, though similar, adolescent cohort. Regardless, this formative period, prior to adulthood, may ultimately offer an opportunity for targeted microbiome inventions. Future work should seek to better understand the potential malleability at this life-stage and whether certain interventions can be more effectively leveraged here to align an individual’s weight-trajectory to a healthy state.

## Materials and methods

### Participants and study design

The devilWASTE study was a sub-study of the Social impact of Physical Activity and nutRition in College (SPARC) study, which sought to analyze relationships between lifestyle factors, weight outcomes, and the social networks of first-year college students.^24^ DevilWASTE participants were recruited from the SPARC cohort from six dorms across three different Arizona State University (ASU) campuses. Participants in devilSPARC were recruited from one dorm on each of the three campuses. The exclusion criteria for devilWASTE included age less than 18 years, certain gastrointestinal conditions such as malabsorptive disease, history of eating disorders, antibiotic use 2–3 months prior to study visits, and current conditions that affect the microbiome including HIV infection, diabetes, or high blood pressure. Inclusion criteria included living in a residence hall at ASU, English speaking, and participation in the SPARC study. Eligible participants provided written informed consent before enrollment. The devilWASTE study and the parent SPARC study were approved by the Arizona State University Institutional Review Board.

### Data collection

Recruitment for devilWASTE took place during the academic year starting in August 2015 and data collection continued through May 2016. All participants were made aware of the study at dormitory floor meetings, after which they provided voluntary, written informed consent. Consent was not obtained until study staff ensured that all questions and concerns had been addressed individually with each potential participant. The study protocol, recruitment, and data collection documents were reviewed and approved by the Arizona State University Institutional Review Board on July 30, 2015 (IRB# 1309009596). Participants provided stool samples at up to three of the four SPARC study time points (beginning and end of fall and spring semesters). In total, we collected 507 stool samples from 272 participants. Due to the expected high attrition rate for longitudinal sampling in a shared living environment (e.g., stigma and high-stress environment), we lost 133 participants to follow-up leaving an *n* of 139 for this analysis. Only individuals who provided at least two stool samples (two samples, *n* = 45; three samples, *n* = 94) at any time throughout the study duration were retained in the present study to support this longitudinal analysis. At each time point, anthropometrics, physical activity, and diet information were collected.

Anthropometrics were obtained by trained research staff using Seca 869 scales (Seca, USA) for weight, Seca 217 stadiometers (Seca, USA) for height, and flexible, tension spring-loaded Gulick measuring tapes for waist circumference (Creative Health Products, USA). These measurements were completed up to three times to ensure accuracy. The two measures that were within 0.5 kg and 0.5 cm for weight and height, respectively, were averaged for final measurements. Participant BMIs were calculated and reported in kg/m^2^.

Questionnaires were used to evaluate lifestyle behaviors. Physical activity was assessed with the Godin-Shephard Leisure-Time Physical Activity Questionnaire which categorizes and quantifies activity into vigorous, moderate, and light physical activity.^81^ Sedentary behavior was assessed with an additional question: “Yesterday, how much time did you spend in front of a screen (excluding time in class and being physically active)?”.^82^ Participants selected a response from a range of zero to six hours. Self-reported dietary intake was reported using the National Cancer Institute Dietary Screener Questionnaire that assesses consumption frequency of key food items and food groups.^83^ This tool does not estimate caloric intake but rather tracks consumption of food group categories such as fruits and vegetables, high-fat and processed foods, added sugar, dietary fiber, and whole-grain intake. Weekly alcohol intake was assessed by the number of drinks consumed weekly by asking: “For each day of the week in the calendar below, indicate the number of alcoholic drinks typically consumed on that day (Only if yes to alcohol is selected).” The dropdown ranged from 1 to 15 drinks for all days of the week.

### Fecal sample collection and processing

After anthropometrics and assessments were completed, participants were given a stool sample collection kit (Commode Specimen Collection Kit, Fisher Scientific, Anthem, AZ) and a brief demonstration on how to properly collect a sample. This kit was labeled with a devilWASTE-specific participant ID as well as contact information for the study staff and written instructions. Contents of the kit included a collection bowl and bag that were pre-weighed as well as a cooler and ice pack to keep the sample cold until it reached the Healthy Lifestyles Research Center on the Downtown ASU campus. Participants were instructed to call research staff immediately after collection, so that samples could be retrieved within 60 minutes to avoid bacterial growth and changes in microbial communities. Fecal samples were then stored at −80°C until extraction. Samples were processed after defrosting at 4°C. Between 0.150 and 0.250 g of feces was put into 2 mL PowerBead tubes from a MoBio Power Soil DNA Isolation Kit (12888–100, MoBio, Carlsbad, CA).

MoBio Power Soil DNA Isolation Kits (12888–100, MoBio, Carlsbad, CA) were used to extract microbial DNA from fecal samples in the laboratory of the Principal Investigator (CMW). These kits combine a series of salt and ethanol-based solutions as well as filtering, and centrifugation methods to first decrease the amount of fecal matter in the sample and then break the cell membranes of microbial cells to release the DNA. Due to inherent challenges in extracting DNA from fecal matter which is comprised of a complex mixture of lipids, carbohydrates, salts, and cells, a heating step was incorporated at the beginning of the protocol as well as an additional cleaning protocol to ensure no fecal inhibitors remained in the final DNA sample. These additions are briefly outlined below and were recommended by the extraction kit manufacturer (MoBio, Carlsbad, CA).

The heating step involved heating the PowerBead tube with Bead Solution, fecal sample, and Solution C1 in a heat block at 65°C for 10 min. Heating followed gently inverting the sample tube after the addition of Solution C1. The cleaning protocol involved adding 100 µl of extracted DNA to a new 2 ml collection tube along with 50 µl of Bead Solution, 25 µl of Solution C2, and 25 µl of Solution C3. The sample was vortexed briefly after each reagent addition, followed by centrifugation at 13,000 × g for 2 minutes at room temperature. Supernatant (~160–190 µl) was transferred to a clean 2 ml collection tube. After gently shaking, 400 µl of Solution C4 was added to the collection tube and vortexed to mix. Tube contents were then applied to the spin filter and centrifuged at 10,000 × g for 1 minute at room temperature. Filter flow through was discarded. In two rounds, 500 µl of Solution C5 was added to the spin filter and centrifuged at 10,000 × g for 30 seconds at room temperature. Flow through was discarded and the spin filter was centrifuged at maximum speed for 2 minutes at room temperature. Finally, the DNA was eluted by placing the spin filter in a new 2 ml collection tube, adding 50 µl of Solution C6 to the center of the filter membrane, incubating for 1 minute at room temperature, and centrifuging at room temperature for 1 minute at 10,000 × g, after which the spin filter was discarded.

Once the DNA was isolated, sample quality and concentration were obtained using a QIAxpert high-speed microfluidic UV/VIS spectrophotometer (9002340, QIAGEN, Germantown, MD). A total of 507 longitudinally obtained samples from 272 participants were extracted and sent for sequencing.

### Fecal microbiome analysis

Samples were sequenced at the ASU Biodesign Institute Genomics Core Lab. At the lab, sequences were quantified using the Quant-iT PicoGreen assay (P7589, Invitrogen, Carlsbad, CA). Sequencing methods began with amplification through triplicate PCR in 96-well plates followed by next-generation sequencing to identify bacterial features. Amplification of the 16S rRNA gene sequence was completed using primers for the conserved V4 region of the bacterial genome. The V4 region was identified using the forward 515F primers and 806 R reverse primers containing Illumina adaptor sequences. Purification and quantification materials used for PCR in the Genomics Core Lab included the QIAquick PCR Purification (28106, Qiagen, Germantown, MD), and KAPA Library Quantification Kits (KK4824, Kapa Biosystems, Wilmington, MA). After PCR completion, sequencing was carried out on the Illumina MiSeq instrument (SY-410-1003, Illumina, Inc., San Diego, CA). All protocols were completed in accordance with best practices established by the Earth Microbiome Project guidelines.^84^

The 16S rRNA sequencing produced 7,804,945 reads with a median of 20,093 per sample. Paired end demultiplexed sequences were added into the Quantitative Insights into Microbial Ecology 2 (QIIME2) software pipeline and denoised using the DADA2 command (trimmed at position 14 and truncated at position 250) to remove low-quality regions and construct a feature table using ASVs.^85^ Next, the ASV feature table was passed through the feature-classifier plugin, which was implemented using a naive Bayes machine-learning classifier, pre-trained to discern taxonomy mapped to the latest version of the rRNA database SILVA (138.1; 99% ASVs from 515F/806 R region of sequences).^86^ A phylogenetic tree was then constructed using the fragment-insertion plugin with SILVA at a p-sampling depth of the rarefaction threshold to impute high-quality reads and normalize for uneven sequencing depth between samples. A *phyloseq* (v1.38.0.) object was then created, and downstream analyses and visualizations were performed in R (v4.1.2.). Sequences identified as Archaea and unclassified Bacteria at the Phyla level, as well as mitochondrial and plant DNA, were removed. Samples were rarified for downstream diversity analysis (7,300 sequences/sample).

For the diversity analysis, we implemented a phylogenetically informed approach. Specifically, alpha diversity was measured using Faith’s PD using the *picante* package (v1.8.2.). In addition, the observed ASVs were calculated to survey the overall richness with the *phyloseq* package. Beta diversity was calculated using weighted UniFrac, placing emphasis on dominant taxa, and unweighted UniFrac, to consider dissimilarity merely on membership. These beta diversity metrics were calculated with the *phyloseq* package.

To assess the community type of each participant sample at the genus level, we applied LDA probabilistic models. A low cutoff for taxa filtering was used (i.e., taxa with a total relative abundance of 1e-5 were filtered out) per the nature of this method, as previously described.^55^ Samples were then surveyed to estimate the most preferable number of latent community types using the ‘FindTopicsNumber’ function in the *ldatuning* package (v1.0.2.). The scoring algorithms “CaoJuan2009”^87^ and “Arun2010”^88^ were specified as metrics, and the model fit was established using Gibbs’ sampling method. The number of ideal community types was determined to be 30, and per-community type-per-taxa and per-sample-per-community type were extracted after performing the LDA using the ‘LDA’ function in the *topicsmodels* package (v0.2.12.). The per-sample-per-community type probabilities were multiplied by the read count for each sample. Hierarchical cluster analysis was then performed using an agglomerative nesting approach (based on a lower number of community types) on scale normalized data, as previously reported.^55^ This was conducted with the ‘agnes’ function in the *cluster* package (v2.1.4.) using Ward’s minimum variance method. The optimal number of clusters was determined with the Dunn Index. Visualization was expressed with a dendrogram and cluster plot with the *factoextra* package (v1.0.7).

### Statistical analysis

Anthropometric, behavioral, and dietary data from participants were first assessed for normality using QQ-plots and Shapiro–Wilk’s test. Differences in baseline values between weight change categories were then analyzed by ANOVA or Kruskal–Wallis tests, depending on normality. Associations between these factors were assessed with Spearman’s rank correlation coefficients. Changes in anthropometric, behavioral, and dietary data were calculated for the first and last collection and assessed between weight change categories by ANOVA or Kruskal–Wallis tests with Bonferroni post-hoc comparisons, where appropriate. The association between change in body weight and waist circumference was assessed by Pearson’s partial correlation analysis, controlling for baseline BMI, sex, and individual participant.

For the gut microbiome, alpha diversity metrics were assessed for normality (Shapiro–Wilk’s tests) and log-transformed. Next, LME models were used to test the effect of time and the interaction of group and time using the *nLME* package (v3.1.153.). Models included baseline BMI and sex as covariates with participant included as a random effect. For any significant interactions, pairwise testing was performed using the *emmeans* package (v1.8.0.). Associations between baseline alpha diversity and body weight were assessed with multiple regression models using the *lmtest* package (v0.9.40.) to explore potential differential diet responses using the gut microbiome as a determinant. These associations were calculated using baseline alpha diversity metrics and change in body weight (post – pre values), accounting for sex, baseline BMI, and the individual. For beta diversity, the mantel test was utilized to assess the correlation between the weighted and unweighted UniFrac distance matrices for all samples via the *vegan* package (v2.6.2.) with 999 permutations. Next, PERMANOVA tests were constructed for UniFrac distances in the *vegan* package testing the effects of the individual (nested factor), weight change category, all time points, and the interaction between these factors, controlling for sex and baseline BMI (number of permutations = 999). Contributions from dominant genera to the PERMANOVA models of weighted UniFrac distances were determined by passing the resulting PERMANOVA object through the coefficients function found in the base stats package in R. Intra- and inter-individual compositional variability was calculated between all sequential microbiome samples for both weighted and unweighted UniFrac distances, log transformed, and compared by weight change categories using LME models accounting for baseline BMI, sex, and the individual (as a random effect). To assess the overall differences between intra- and inter-individual variability, all values were pooled by comparison and assessed via a Wilcoxon rank sum test.

To calculate the ratio between the genera *Prevotella* and *Bacteroides*, we converted taxonomic data to relative abundance, added a pseudo count of 5.0 × 10^−7^ to samples where *Prevotella* was not present (as previously described),^37^ performed a log10 transformation on both, and then divided *Prevotella* by *Bacteroides*. Employing the *nLME* package a LME model was used to test the effect of time (first and last time points) and the interaction of group and time with baseline BMI and sex as covariates with participant included as a random effect. For any significant interactions, pairwise testing was performed using the *emmeans* package. The PSI was calculated for baseline CLR transformed relative abundance of *Prevotella* and *Bacteroides* using the ‘bimodality’ function in the *microbiome* package (v1.18.0.). A TP analysis was calculated on the CLR transformed relative abundance of *Prevotella* and *Bacteroides* for all samples using a bootstrapped potential analysis (*n* bootstrap iterations = 100) with the *microbiome* package. The abundance deviation of these genera for each participant was summed and compared by participants that crossed the TP and those that did not using Wilcoxon rank sum tests. To assess the trend of state transition between *Prevotella* and *Bacteroides*, we performed polynomial regression with the *nLME* package using the difference in their relative abundance and their sum subtracted from one, as previously described.^33^

Variation of taxa at the genus level was assessed for all samples on CLR-transformed relative abundance of features with a detection of ≥1.0% for at least 10% of the samples. Intra- and inter-individual variance was extracted for each taxon via LMM, with participant acting as a random variable using the function ‘lmer’ in the package *Lme4* (v1.1.30.). Total variance was calculated by summing the intra- and inter-individual variance and ICC by dividing the inter-individual variation by the total variation of the relative abundance for each genus. Associations between CLR relative abundance and ICC and total variation were performed using Spearman’s rank correlation tests. Differences between weight change categories for ICC and total variance were performed using Wilcoxon rank sum tests.

For the LDA data, associations between predictor variables and community types were assessed using Dirichlet regression models with the *DirichletReg* package (v0.7.1). Models were first constructed for weight change trajectory with baseline samples of the 30 latent community types as the response variables. Next, the second and third longitudinal samples were pooled, and models were formed for 13 predictors as continuous variables (anthropometric, dietary, and behavioral factors) with the 30 community types. All predictor variables were standardized by their standard deviation, as previously described^55^. Model fit was assessed by building in relevant covariates, which ultimately led to each model being adjusted for sex and initial BMI. An alpha value of <0.05 was used to denote statistical significance, and *P*-value adjustments were performed where appropriate using the Benjamini–Hochberg (BH) procedure. All statistical analyses were performed in the R environment (v4.1.2.).

## Supplementary Material

Supplemental Material

## Data Availability

Due to the vulnerable nature of adolescent and emerging adult populations, researchers did not seek participant consent to share study data. Therefore, participants of this study did not agree for their data to be shared publicly, and supporting data are not available.
