# Supplementary material for: Gut microbiome diversity, variability, and latent community types compared with shifts in body weight during the freshman year of college in dormitory-housed adolescents
Source: Gut Microbes. 2023 Aug 29;15(2):2250482. doi: 10.1080/19490976.2023.2250482 (PMC10467528; doi:10.1080/19490976.2023.2250482)
Supplement: Supplemental Material [file KGMI_A_2250482_SM4851.docx]

**Supplemental Material**

| Table 1 Baseline lifestyle and dietary behaviors of study participants | | | | | |
| --- | --- | --- | --- | --- | --- |
| Behavior | **Total (*n*=139)** | **WG (*n*=67)** | **WL (*n*=13)** | **WM (*n*=59)** | ***P*-value** |
| Screen time (minutes per week)  categorical, % (*n*)  0-75  195  315-360 | 26.6 (37)  35.3 (49)  38.1 (53) | 26.9 (18)  31.3 (21)  41.8 (28) | 30.8 (4)  30.8 (4)  38.5 (5) | 25.4 (15)  40.7 (24)  33.9 (20) | 0.261 |
| Moderate to vigorous physical activity, %  (*n*)  Active (score of ≥ 24)  Moderately active (score  of 14-23)  Sedentary (score < 14) | 44.8 ± 30.9  64.7 (90)  18.0 (25)  17.3 (24) | 48.7 ± 31.4  70.1 (47)  14.9 (10)  14.9 (10) | 38.2 ± 28.9  69.2 (9)  7.7 (1)  23.1 (3) | 41.7 ± 30.7  57.6 (34)  23.7 (14)  18.6 (11) | 0.338 |
| Depression score | 1.99 ± 0.76 | 1.96 ± 0.77 | 1.63 ± 0.54 | 2.15 ± 0.82 | 0.091 |
| Hours of nightly sleep^a^ | 7.4 ± 0.9 | 7.5 ± 1.0 | 7.4 ± 1.0 | 7.4 ± 0.9 | 0.583 |
| Alcohol intake (No.  beverages over the last  7 days) | 3.29 ± 5.41 | 4.04 ± 6.40 | 2.31 ± 2.95 | 2.56 ± 4.38 | 0.576 |
| Fruit/vegetable intake^b^ | 2.4 ± 0.8 | 2.5 ± 0.9 | 2.5 ± 0.8 | 2.4 ± 0.9 | 0.532 |
| Whole grain intake^b^ | 0.7 ± 0.3 | 0.8 ± 0.5 | 0.6 ± 0.4 | 0.7 ± 0.2 | 0.120 |
| Dairy intake^b^ | 1.9 ± 0.8 | 2.1 ± 1.3 | 1.6 ± 0.6 | 1.8 ± 0.8 | 0.224 |
| Red/processed meat  intake^c^ | 0.8 ± 0.7 | 0.9 ± 0.8 | 0.5 ± 0.4 | 0.7 ± 0.8 | 0.128 |
| Daily sugar intake (g) | 18.1 ± 7.7 | 19.6 ± 15.8 | 22.1 ± 15.3 | 17.5 ± 7.3 | 0.298 |
| Daily fiber intake (g) | 15.7 ± 3.6 | 16.5 ± 4.6 | 15.4 ± 4.7 | 15.4 ± 3.6 | 0.263 |
| Data displayed as mean ± SD, unless stated otherwise. Abbreviations: WG, weight gain (gain of >3% of baseline weight); WL, weight loss (loss of >3% body weight); WM, weight maintenance (less than or equal to ±3% weight change).  Superscripts: ^a^Average of combined weekday and weekend nightly sleep hours; ^b^Expressed as daily cup equivalents; ^c^Daily number of servings. | | | | | |

| Table S2 Absolute and relative percent change for anthropometric outcomes of the three weight classifications | | | |
| --- | --- | --- | --- |
| Outcome | **WG (*n*=67)** | **WL (*n*=13)** | **WM (*n*=59)** |
| Weight | 4.59 ± 2.61 kg* | -5.02 ± 2.34 kg* | 0.51 ± 1.16 kg |
|  | 6.61 ± 2.98%* | -7.06 ± 2.99%* | 0.76 ± 1.57% |
| Waist circumference | 4.19 ± 5.11 cm* | -4.07 ± 4.31 cm* | -0.14 ± 4.28 cm |
|  | 5.44 ± 6.67%* | -4.44 ± 5.06% | -0.01 ± 5.39% |
| Waist/hip ratio | 0.02 ± 0.05 cm* | -0.01 ± 0.05 cm | <0.01 ± 0.04 cm |
|  | 2.91 ± 6.03% | -1.04 ± 5.21% | 0.26 ± 5.55% |
| BMI | 1.51 ± 0.86 kg/m^2^ | -1.88 ± 0.81 kg/m^2^ | 0.14 ± 0.45 kg/m^2^ |
|  | 6.22 ± 3.21% | -7.49 ± 2.91% | 0.58 ± 1.76% |
| Data displayed as mean ± SD, unless stated otherwise. Abbreviations: WG, weight gain (gain of >3% of baseline weight); WL, weight loss (loss of >3% body weight); WM, weight maintenance (less than or equal to ±3% weight change); BMI, body mass index.  **P* < 0.05 vs. WM. | | | |

| Table S3 Change (Δ) in lifestyle behavior outcomes for the three weight classifications | | | | |
| --- | --- | --- | --- | --- |
| Outcome | **WG (*n*=67)** | **WL (*n*=13)** | **WM (*n*=59)** | ***P*-value** |
| Δ Moderate to vigorous physical activity (min) | -4.86 ± 34.89 | -4.45 ± 37.89 | 4.43 ± 27.64 | 0.254 |
| Δ Depression score | 0.06 ± 0.92 | 0.12 ± 0.78 | 0.14 ± 0.76 | 0.656 |
| Δ Hours of nightly sleep^a^ | -0.28 ± 1.39 | 0.03 ± 0.76 | -0.29 ± 0.99 | 0.481 |
| Δ Alcohol intake (No. beverages over the last 7 days) | -0.21 ± 5.16 | -0.23 ± 1.59 | -0.11 ± 3.66 | 0.824 |
| Δ Fruit/vegetable intake^b^ | -0.15 ± 0.67 | -0.17 ± 0.76 | -0.18 ± 0.83 | 0.976 |
| Δ Whole grain intake^b^ | -0.06 ± 0.37 | 0.05 ± 0.53 | 0.02 ± 0.31 | 0.209 |
| Δ Dairy intake^b^ | -0.41 ± 0.69 | -0.29 ± 0.44 | -0.21 ± 0.59 | 0.280 |
| Δ Red/processed meat intake^c^ | -0.20 ± 0.86 | -0.19 ± 0.31 | -0.13 ± 0.71 | 0.460 |
| Δ Daily sugar intake (g) | -1.21 ± 7.11 | -7.07 ± 16.79 | -1.25 ± 7.61 | 0.813 |
| Δ Daily fiber intake (g) | -0.64 ± 3.32 | -0.29 ± 4.28 | -0.48 ± 2.82 | 0.828 |
| Data displayed as mean ± SD. Abbreviations: WG, weight gain (gain of >3% of baseline weight); WL, weight loss (loss of >3% body weight); WM, weight maintenance (less than or equal to ±3% weight change).  Superscripts: ^a^Average of combined weekday and weekend nightly sleep hours; ^b^Expressed as daily cup equivalents; ^c^Daily number of servings. | | | | |

| Table S4 Unweighted UniFrac PERMANOVA model and term results | | | | | | |
| --- | --- | --- | --- | --- | --- | --- |
|  | **DF** | **SS** | **R^2^** | **F Model** | **Pr(>F)** | **P.Adj** |
| Individual | 138 | 47.188 | 0.605 | 3.163 | 0.001 | 0.002 |
| Weight cluster: Time | 3 | 0.388 | 0.005 | 1.196 | 0.075 | 0.075 |
| Weight cluster: Time: Individual | 136 | 20.235 | 0.259 | 1.376 | 0.001 | 0.002 |
| Residuals | 94 | 10.163 | 0.130 |  |  |  |
| Total | **371** | **77.974** | **1.000** |  |  |  |
|  | | | | | | |

| Table S5 Weighted UniFrac PERMANOVA model and term results | | | | | | |
| --- | --- | --- | --- | --- | --- | --- |
|  | **DF** | **SS** | **R^2^** | **F Model** | **Pr(>F)** | **P.Adj** |
| Individual | 138 | 6.836 | 0.606 | 3.254 | <0.001 | 0.002 |
| Weight cluster: Time | 3 | 0.120 | 0.011 | 2.636 | 0.003 | 0.003 |
| Weight cluster: Time: Individual | 136 | 2.897 | 0.257 | 1.399 | <0.001 | 0.002 |
| Residuals | 94 | 1.431 | 0.127 |  |  |  |
| Total | **371** | **11.284** | **1.000** |  |  |  |
|  | | | | | | |

| **Table S6** Relative abundance, center log-ratio (CLR) relative abundance, intra- and inter-individual variability, total variance, and intraclass correlation coefficients (ICC) of taxa for all study samples at the genus level. | | | | | | | |
| --- | --- | --- | --- | --- | --- | --- | --- |
| **Genus** | **Relative Abundance** | **CLR Relative Abundance** | **Intra-individual Variation** | **Inter-individual Variation** | **Total Variance** | **ICC** | **>0.50 ICC?** |
| *Agathobacter* | 0.061 | 3.493 | 2.123 | 2.824 | 4.947 | 0.571 | Y |
| *Akkermansia* | 0.002 | -1.604 | 1.468 | 3.482 | 4.950 | 0.703 | Y |
| *Alistipes* | 0.033 | 2.332 | 2.618 | 3.128 | 5.746 | 0.544 | Y |
| *Anaerostipes* | 0.021 | 2.923 | 0.977 | 1.164 | 2.141 | 0.544 | Y |
| *Bacteroides* | 0.203 | 5.204 | 0.949 | 1.419 | 2.368 | 0.599 | Y |
| *Barnesiella* | 0.004 | -1.303 | 4.225 | 2.123 | 6.348 | 0.334 | **N** |
| *Bifidobacterium* | 0.077 | 3.124 | 5.697 | 3.352 | 9.049 | 0.370 | **N** |
| *Bilophila* | 0.001 | -1.654 | 0.780 | 2.064 | 2.844 | 0.726 | Y |
| *Blautia* | 0.106 | 4.813 | 0.490 | 0.712 | 1.202 | 0.592 | Y |
| *Butyricicoccus* | 0.004 | 0.491 | 1.198 | 3.486 | 4.684 | 0.744 | Y |
| *CAG 352* | 0.021 | 0.290 | 4.737 | 5.146 | 9.883 | 0.521 | Y |
| *CAG 56* | 0.001 | -1.559 | 2.643 | 1.829 | 4.472 | 0.409 | **N** |
| *Christensenellaceae R7 group* | 0.011 | 0.740 | 3.207 | 2.351 | 5.558 | 0.423 | **N** |
| *Clostridia UCG 014* | 0.003 | -1.939 | 2.593 | 1.973 | 4.566 | 0.432 | **N** |
| *Clostridium sensu stricto 1* | 0.003 | -0.595 | 1.040 | 4.012 | 5.052 | 0.794 | Y |
| *Colidextribacter* | 0.001 | -1.523 | 0.864 | 1.817 | 2.681 | 0.678 | Y |
| *Collinsella* | 0.009 | 0.438 | 4.321 | 2.661 | 6.982 | 0.381 | **N** |
| *Coprococcus* | 0.010 | 1.555 | 2.570 | 1.549 | 4.119 | 0.376 | **N** |
| *Desulfovibrio* | 0.001 | -2.200 | 1.432 | 1.504 | 2.936 | 0.512 | Y |
| *Dorea* | 0.021 | 2.508 | 2.529 | 1.756 | 4.285 | 0.410 | **N** |
| *DTU089* | 0.000 | -2.009 | 0.923 | 1.879 | 2.803 | 0.671 | Y |
| *Escherichia Shigella* | 0.007 | -0.772 | 2.699 | 4.301 | 7.000 | 0.614 | Y |
| *Eubacterium coprostanoligenes group* | 0.012 | 0.670 | 3.200 | 3.596 | 6.796 | 0.529 | Y |
| *Eubacterium eligens group* | 0.001 | -1.580 | 0.744 | 3.369 | 4.113 | 0.819 | Y |
| *Eubacterium hallii group* | 0.022 | 2.649 | 1.784 | 1.993 | 3.777 | 0.528 | Y |
| *Eubacterium siraeum group* | 0.003 | -1.594 | 1.634 | 2.697 | 4.331 | 0.623 | Y |
| *Eubacterium ventriosum group* | 0.002 | -0.449 | 1.118 | 2.844 | 3.962 | 0.718 | Y |
| *Eubacterium xylanophilum group* | 0.001 | -1.993 | 0.677 | 2.176 | 2.853 | 0.763 | Y |
| *Faecalibacterium* | 0.094 | 4.229 | 1.430 | 1.462 | 2.892 | 0.506 | Y |
| *Flavonifractor* | 0.002 | -0.645 | 2.009 | 3.496 | 5.505 | 0.635 | Y |
| *Fusicatenibacter* | 0.016 | 2.305 | 2.003 | 1.834 | 3.837 | 0.478 | **N** |
| *Haemophilus* | 0.003 | -1.678 | 1.083 | 4.283 | 5.366 | 0.798 | Y |
| *Holdemanella* | 0.004 | -2.462 | 2.325 | 1.588 | 3.913 | 0.406 | **N** |
| *Incertae Sedis* | 0.010 | 1.924 | 0.593 | 1.426 | 2.020 | 0.706 | Y |
| *Lachnoclostridium* | 0.007 | 0.951 | 2.292 | 3.435 | 5.727 | 0.600 | Y |
| *Lachnospira* | 0.002 | -1.034 | 0.693 | 4.102 | 4.795 | 0.856 | Y |
| *Lachnospiraceae FCS020 group* | 0.001 | -1.476 | 0.965 | 2.073 | 3.038 | 0.682 | Y |
| *Lachnospiraceae ND3007 group* | 0.001 | -1.366 | 1.372 | 2.553 | 3.925 | 0.650 | Y |
| *Lachnospiraceae NK4A136 group* | 0.009 | 1.203 | 1.731 | 2.881 | 4.612 | 0.625 | Y |
| *Lachnospiraceae UCG 001* | 0.001 | -2.077 | 0.947 | 2.205 | 3.152 | 0.700 | Y |
| *Lachnospiraceae UCG 004* | 0.000 | -2.015 | 0.558 | 2.749 | 3.307 | 0.831 | Y |
| *Marvinbryantia* | 0.001 | -1.721 | 0.941 | 2.284 | 3.224 | 0.708 | Y |
| *Monoglobus* | 0.006 | 1.244 | 0.912 | 2.654 | 3.566 | 0.744 | Y |
| *NK4A214 group* | 0.001 | -1.352 | 1.450 | 1.693 | 3.143 | 0.539 | Y |
| *Oscillibacter* | 0.001 | -1.606 | 0.838 | 2.292 | 3.130 | 0.732 | Y |
| Other | 0.017 | 2.306 | 0.794 | 1.241 | 2.034 | 0.610 | Y |
| *Parabacteroides* | 0.010 | 1.123 | 1.529 | 3.366 | 4.895 | 0.688 | Y |
| *Parasutterella* | 0.004 | -1.131 | 3.532 | 3.037 | 6.569 | 0.462 | **N** |
| *Phascolarctobacterium* | 0.002 | -1.373 | 2.169 | 2.062 | 4.231 | 0.487 | **N** |
| *Prevotella* | 0.036 | -1.052 | 6.510 | 3.393 | 9.903 | 0.343 | **N** |
| *Roseburia* | 0.009 | 1.200 | 1.391 | 3.431 | 4.822 | 0.712 | Y |
| *Ruminococcus* | 0.008 | 0.508 | 2.847 | 3.079 | 5.926 | 0.520 | Y |
| *Ruminococcus gauvreauii group* | 0.008 | 0.834 | 3.405 | 2.920 | 6.325 | 0.462 | **N** |
| *Ruminococcus gnavus group* | 0.006 | -0.879 | 5.650 | 3.526 | 9.176 | 0.384 | **N** |
| *Ruminococcus torques group* | 0.012 | 2.198 | 0.846 | 1.876 | 2.722 | 0.689 | Y |
| *Sellimonas* | 0.001 | -2.190 | 2.512 | 1.511 | 4.023 | 0.376 | **N** |
| *Subdoligranulum* | 0.051 | 3.366 | 2.592 | 1.941 | 4.533 | 0.428 | **N** |
| *Sutterella* | 0.001 | -1.969 | 1.424 | 2.216 | 3.640 | 0.609 | Y |
| *Turicibacter* | 0.003 | -0.260 | 1.112 | 3.637 | 4.749 | 0.766 | Y |
| *Tyzzerella* | 0.001 | -2.294 | 2.491 | 1.700 | 4.191 | 0.406 | **N** |
| *UBA1819* | 0.002 | -0.364 | 1.134 | 2.442 | 3.576 | 0.683 | Y |
| *UCG 002* | 0.010 | 0.785 | 2.994 | 2.136 | 5.130 | 0.416 | **N** |
| *UCG 003* | 0.001 | -1.306 | 1.009 | 2.058 | 3.067 | 0.671 | Y |
| *UCG 005* | 0.002 | -1.102 | 1.899 | 1.997 | 3.896 | 0.513 | Y |
| *UCG 010* | 0.001 | -2.163 | 0.926 | 1.389 | 2.315 | 0.600 | Y |
| Unclassified Lachnospiraceae | 0.012 | 2.252 | 1.012 | 1.308 | 2.320 | 0.564 | Y |
| Unclassified Oscillospiraceae | 0.000 | -2.505 | 1.254 | 0.706 | 1.960 | 0.360 | **N** |
| Unclassified Ruminococcaceae | 0.001 | -1.765 | 0.765 | 2.557 | 3.322 | 0.770 | Y |
| Uncultured | 0.005 | 0.898 | 0.889 | 2.537 | 3.426 | 0.741 | Y |

| **Table S7** Center log-ratio (CLR) relative abundance, intra- and inter-individual variability, and intraclass correlation coefficients (ICC) of taxa at the genus level for each weight change trajectory. | | | | | | | | | | | | |
| --- | --- | --- | --- | --- | --- | --- | --- | --- | --- | --- | --- | --- |
|  | **CLR Relative Abundance** | | | **Intra-individual Variation** | | | **Inter-individual Variation** | | | **ICC** | | |
| **Genus** | **WG** | **WL** | **WM** | **WG** | **WL** | **WM** | **WG** | **WL** | **WM** | **WG** | **WL** | **WM** |
| *Agathobacter* | 3.552 | 3.056 | 3.524 | 1.825 | 1.849 | 2.577 | 3.058 | 3.948 | 2.351 | 0.626 | 0.681 | 0.477 |
| *Akkermansia* | -1.738 | -1.817 | -1.399 | 1.978 | 0.000 | 1.360 | 2.307 | 6.486 | 3.961 | 0.538 | 1.000 | 0.744 |
| *Alistipes* | 2.347 | 1.881 | 2.417 | 1.920 | 4.918 | 2.967 | 3.290 | 2.508 | 3.074 | 0.631 | 0.338 | 0.509 |
| *Anaerostipes* | 3.037 | 2.355 | 2.917 | 1.231 | 0.535 | 0.707 | 1.033 | 1.077 | 1.351 | 0.456 | 0.668 | 0.656 |
| *Bacteroides* | 5.129 | 4.946 | 5.348 | 1.562 | 0.356 | 0.355 | 1.460 | 0.934 | 1.475 | 0.483 | 0.724 | 0.806 |
| *Barnesiella* | -1.056 | -0.438 | -1.783 | 5.219 | 4.184 | 1.676 | 1.624 | 3.417 | 1.551 | 0.237 | 0.450 | 0.481 |
| *Bifidobacterium* | 3.208 | 2.998 | 3.056 | 6.386 | 3.500 | 5.583 | 2.811 | 3.096 | 4.010 | 0.306 | 0.469 | 0.418 |
| *Bilophila* | -1.736 | -1.294 | -1.639 | 0.808 | 1.501 | 0.648 | 2.063 | 2.273 | 1.989 | 0.719 | 0.602 | 0.754 |
| *Blautia* | 4.936 | 4.309 | 4.784 | 0.571 | 0.531 | 0.332 | 0.788 | 0.431 | 0.679 | 0.580 | 0.448 | 0.672 |
| *Butyricicoccus* | 0.483 | 0.648 | 0.466 | 1.445 | 0.130 | 1.183 | 3.446 | 2.991 | 3.714 | 0.705 | 0.958 | 0.758 |
| *CAG 352* | 0.433 | 0.294 | 0.124 | 6.227 | 2.731 | 3.666 | 4.112 | 6.669 | 6.084 | 0.398 | 0.709 | 0.624 |
| *CAG 56* | -1.329 | -1.881 | -1.753 | 3.162 | 2.150 | 2.143 | 1.466 | 1.998 | 2.200 | 0.317 | 0.482 | 0.507 |
| *Christensenellaceae R7 group* | 0.678 | 1.245 | 0.700 | 2.802 | 3.717 | 3.521 | 2.319 | 1.349 | 2.695 | 0.453 | 0.266 | 0.434 |
| *Clostridia UCG 014* | -2.159 | -1.309 | -1.824 | 1.489 | 4.627 | 3.333 | 2.277 | 3.157 | 1.366 | 0.605 | 0.406 | 0.291 |
| *Clostridium sensu stricto 1* | -0.632 | -0.515 | -0.571 | 1.198 | 2.346 | 0.622 | 3.703 | 2.341 | 4.758 | 0.756 | 0.499 | 0.884 |
| *Colidextribacter* | -1.656 | -1.198 | -1.441 | 1.045 | 1.563 | 0.459 | 1.447 | 2.135 | 2.199 | 0.581 | 0.577 | 0.827 |
| *Collinsella* | 0.528 | 0.753 | 0.263 | 3.861 | 3.170 | 5.171 | 2.822 | 2.742 | 2.496 | 0.422 | 0.464 | 0.326 |
| *Coprococcus* | 1.508 | 1.385 | 1.648 | 3.465 | 1.095 | 1.885 | 1.192 | 2.024 | 1.889 | 0.256 | 0.649 | 0.501 |
| *Desulfovibrio* | -2.214 | -1.492 | -2.342 | 1.605 | 2.474 | 0.838 | 0.979 | 2.511 | 1.964 | 0.379 | 0.504 | 0.701 |
| *Dorea* | 2.408 | 2.424 | 2.642 | 3.189 | 0.445 | 2.324 | 1.906 | 2.420 | 1.409 | 0.374 | 0.845 | 0.377 |
| *DTU089* | -2.092 | -2.569 | -1.786 | 1.020 | 0.288 | 0.848 | 1.430 | 2.370 | 2.302 | 0.584 | 0.892 | 0.731 |
| *Escherichia Shigella* | -0.704 | -0.896 | -0.823 | 2.268 | 1.520 | 3.455 | 5.278 | 2.672 | 3.618 | 0.699 | 0.637 | 0.512 |
| *Eubacterium coprostanoligenes group* | 0.495 | 1.223 | 0.748 | 3.880 | 5.791 | 1.881 | 2.939 | 1.809 | 4.736 | 0.431 | 0.238 | 0.716 |
| *Eubacterium eligens group* | -1.674 | -1.723 | -1.439 | 0.702 | 0.747 | 0.824 | 3.049 | 4.893 | 3.388 | 0.813 | 0.868 | 0.804 |
| *Eubacterium hallii group* | 2.518 | 2.365 | 2.864 | 2.739 | 0.235 | 1.060 | 2.182 | 2.577 | 1.622 | 0.443 | 0.916 | 0.605 |
| *Eubacterium siraeum group* | -1.656 | -1.641 | -1.511 | 1.994 | 0.911 | 1.448 | 2.708 | 3.117 | 2.574 | 0.576 | 0.774 | 0.640 |
| *Eubacterium ventriosum group* | -0.535 | -0.846 | -0.260 | 1.266 | 0.965 | 0.977 | 2.634 | 2.861 | 3.120 | 0.675 | 0.748 | 0.761 |
| *Eubacterium xylanophilum group* | -1.977 | -2.255 | -1.953 | 0.709 | 1.227 | 0.559 | 1.992 | 2.424 | 2.322 | 0.737 | 0.664 | 0.806 |
| *Faecalibacterium* | 4.250 | 4.158 | 4.221 | 1.843 | 1.232 | 1.046 | 1.454 | 3.308 | 1.057 | 0.441 | 0.729 | 0.503 |
| *Flavonifractor* | -0.782 | -1.574 | -0.277 | 2.080 | 1.096 | 1.908 | 3.496 | 3.799 | 3.397 | 0.627 | 0.776 | 0.640 |
| *Fusicatenibacter* | 2.166 | 2.204 | 2.490 | 2.395 | 0.548 | 1.856 | 1.855 | 1.885 | 1.846 | 0.436 | 0.775 | 0.499 |
| *Haemophilus* | -1.582 | -1.978 | -1.722 | 0.336 | 3.509 | 1.448 | 5.006 | 2.700 | 3.783 | 0.937 | 0.435 | 0.723 |
| *Holdemanella* | -2.438 | -2.758 | -2.423 | 2.627 | 0.626 | 2.356 | 1.025 | 2.694 | 1.998 | 0.281 | 0.811 | 0.459 |
| *Incertae_Sedis* | 2.007 | 1.468 | 1.930 | 0.374 | 0.577 | 0.808 | 1.351 | 2.854 | 1.212 | 0.783 | 0.832 | 0.600 |
| *Lachnoclostridium* | 1.101 | 0.154 | 0.956 | 2.417 | 2.267 | 2.147 | 2.948 | 5.993 | 3.383 | 0.549 | 0.726 | 0.612 |
| *Lachnospira* | -0.937 | -1.035 | -1.146 | 1.096 | 0.765 | 0.281 | 4.052 | 4.460 | 4.038 | 0.787 | 0.854 | 0.935 |
| *Lachnospiraceae FCS020 group* | -1.269 | -1.708 | -1.664 | 0.737 | 0.620 | 1.285 | 2.285 | 1.746 | 1.868 | 0.756 | 0.738 | 0.592 |
| *Lachnospiraceae ND3007 group* | -1.096 | -1.868 | -1.568 | 1.414 | 1.066 | 1.304 | 2.677 | 2.926 | 2.292 | 0.654 | 0.733 | 0.637 |
| *Lachnospiraceae NK4A136 group* | 1.031 | 1.988 | 1.227 | 1.832 | 0.694 | 1.818 | 2.894 | 2.028 | 3.006 | 0.612 | 0.745 | 0.623 |
| *Lachnospiraceae UCG 001* | -1.984 | -1.570 | -2.299 | 1.189 | 1.380 | 0.504 | 2.274 | 2.731 | 2.008 | 0.657 | 0.664 | 0.799 |
| *Lachnospiraceae UCG 004* | -2.002 | -1.721 | -2.095 | 0.893 | 0.345 | 0.248 | 2.531 | 3.647 | 2.780 | 0.739 | 0.914 | 0.918 |
| *Marvinbryantia* | -1.640 | -1.219 | -1.927 | 1.460 | 0.401 | 0.445 | 1.966 | 3.632 | 2.321 | 0.574 | 0.901 | 0.839 |
| *Monoglobus* | 1.318 | 0.555 | 1.312 | 1.224 | 0.862 | 0.598 | 2.602 | 2.553 | 2.712 | 0.680 | 0.748 | 0.819 |
| *NK4A214 group* | -1.478 | -0.695 | -1.354 | 1.203 | 2.238 | 1.542 | 1.755 | 1.761 | 1.581 | 0.593 | 0.440 | 0.506 |
| *Oscillibacter* | -1.639 | -1.586 | -1.573 | 0.656 | 2.096 | 0.841 | 2.476 | 2.796 | 1.966 | 0.791 | 0.572 | 0.700 |
| Other | 2.259 | 2.361 | 2.349 | 0.755 | 0.372 | 0.960 | 1.470 | 1.044 | 1.008 | 0.661 | 0.737 | 0.512 |
| *Parabacteroides* | 1.058 | 1.198 | 1.181 | 1.710 | 1.296 | 1.439 | 3.230 | 2.693 | 3.660 | 0.654 | 0.675 | 0.718 |
| *Parasutterella* | -1.129 | -1.467 | -1.059 | 3.757 | 1.863 | 3.568 | 3.400 | 3.223 | 2.726 | 0.475 | 0.634 | 0.433 |
| *Phascolarctobacterium* | -1.597 | -0.601 | -1.287 | 1.561 | 3.334 | 2.427 | 1.944 | 1.742 | 2.350 | 0.555 | 0.343 | 0.492 |
| *Prevotella* | -0.805 | -0.243 | -1.520 | 6.528 | 4.476 | 6.690 | 2.729 | 8.920 | 2.905 | 0.295 | 0.666 | 0.303 |
| *Roseburia* | 1.412 | 1.337 | 0.924 | 1.386 | 1.286 | 1.342 | 3.387 | 2.243 | 3.741 | 0.710 | 0.636 | 0.736 |
| *Ruminococcus* | 0.628 | 0.503 | 0.371 | 2.707 | 3.708 | 2.974 | 2.853 | 3.462 | 3.224 | 0.513 | 0.483 | 0.520 |
| *Ruminococcus gauvreauii group* | 0.802 | -0.061 | 1.071 | 4.271 | 2.583 | 2.548 | 2.404 | 3.779 | 3.327 | 0.360 | 0.594 | 0.566 |
| *Ruminococcus gnavus group* | -0.860 | -1.979 | -0.656 | 5.783 | 5.193 | 5.495 | 4.114 | 1.642 | 3.229 | 0.416 | 0.240 | 0.370 |
| *Ruminococcus torques group* | 2.246 | 2.062 | 2.173 | 0.793 | 0.524 | 0.997 | 1.661 | 2.454 | 2.018 | 0.677 | 0.824 | 0.669 |
| *Sellimonas* | -2.101 | -3.048 | -2.101 | 2.587 | 0.081 | 2.847 | 1.584 | 1.982 | 1.331 | 0.380 | 0.961 | 0.319 |
| *Subdoligranulum* | 3.427 | 2.811 | 3.420 | 3.180 | 2.657 | 1.803 | 1.456 | 1.703 | 2.625 | 0.314 | 0.391 | 0.593 |
| *Sutterella* | -2.198 | -0.878 | -1.947 | 1.108 | 2.331 | 1.253 | 1.674 | 2.418 | 2.877 | 0.602 | 0.509 | 0.697 |
| *Turicibacter* | -0.284 | -0.378 | -0.205 | 0.924 | 1.565 | 1.236 | 3.685 | 3.848 | 3.624 | 0.799 | 0.711 | 0.746 |
| *Tyzzerella* | -2.113 | -2.593 | -2.436 | 2.728 | 3.046 | 2.138 | 2.058 | 1.673 | 1.271 | 0.430 | 0.355 | 0.373 |
| *UBA1819* | -0.518 | -0.775 | -0.094 | 0.961 | 0.943 | 1.281 | 2.317 | 3.362 | 2.403 | 0.707 | 0.781 | 0.652 |
| *UCG 002* | 0.735 | 1.766 | 0.624 | 2.632 | 2.492 | 3.445 | 2.709 | 1.789 | 1.530 | 0.507 | 0.418 | 0.308 |
| *UCG 003* | -1.456 | -0.734 | -1.261 | 0.777 | 2.448 | 0.950 | 2.131 | 1.516 | 2.065 | 0.733 | 0.382 | 0.685 |
| *UCG 005* | -1.149 | 0.069 | -1.311 | 1.609 | 1.443 | 2.104 | 2.154 | 2.107 | 1.764 | 0.572 | 0.594 | 0.456 |
| *UCG 010* | -2.113 | -1.762 | -2.310 | 0.593 | 1.277 | 1.254 | 1.530 | 2.178 | 1.019 | 0.721 | 0.630 | 0.448 |
| Unclassified Lachnospiraceae | 2.337 | 2.047 | 2.200 | 1.166 | 0.636 | 0.957 | 1.170 | 1.519 | 1.405 | 0.501 | 0.705 | 0.595 |
| Unclassified Oscillospiraceae | -2.532 | -1.332 | -2.737 | 1.210 | 2.240 | 0.799 | 0.721 | 1.726 | 0.448 | 0.373 | 0.435 | 0.359 |
| Unclassified Ruminococcaceae | -1.899 | -1.928 | -1.573 | 0.640 | 1.196 | 0.833 | 2.252 | 3.155 | 2.738 | 0.779 | 0.725 | 0.767 |
| Uncultured | 0.724 | 0.802 | 1.122 | 0.608 | 1.675 | 1.030 | 3.229 | 2.734 | 1.667 | 0.842 | 0.620 | 0.618 |

Abbreviations: WG, weight gain (gain of >3% of baseline weight); WL, weight loss (loss of >3% body weight); WM, weight maintenance (less than or equal to ±3% weight change).


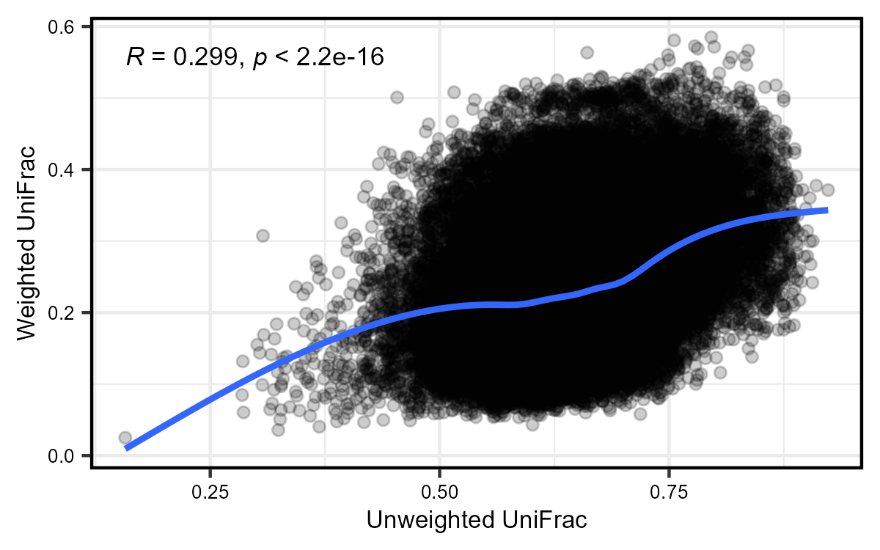


**Figure S1.** Mantel test performed between total weighted and unweighted UniFrac distances from all 139 participants microbiome samples (number of permutations = 999).


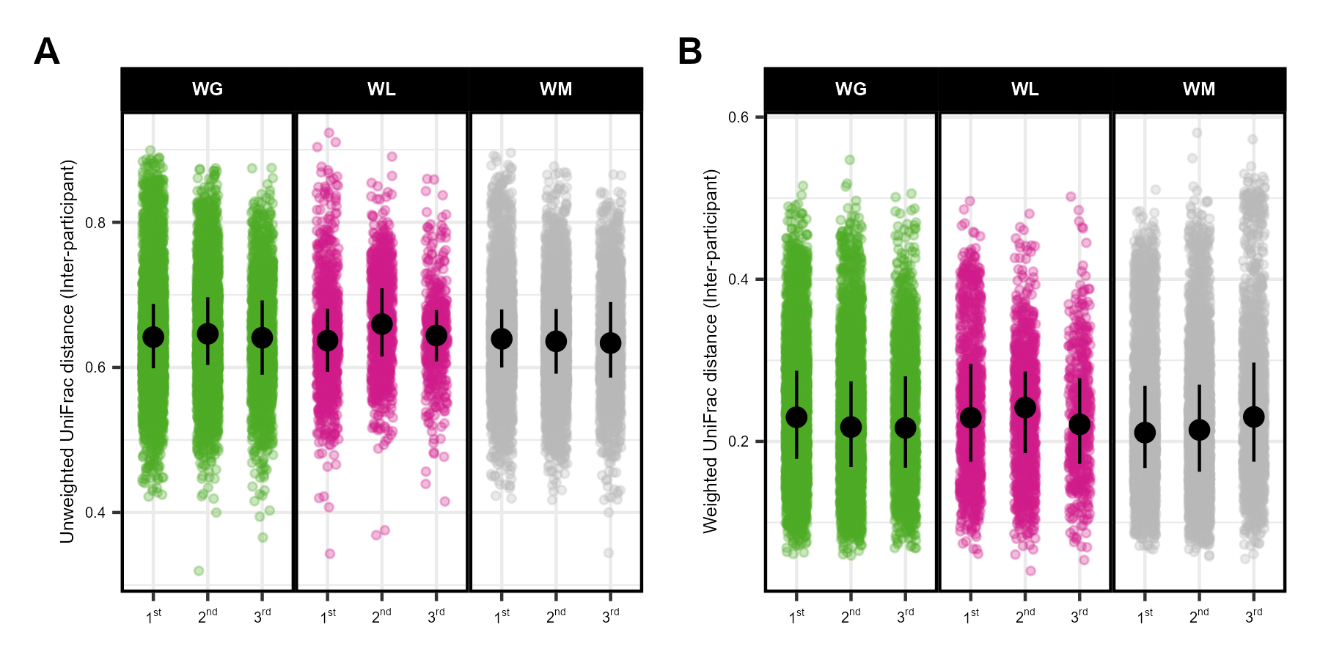


**Figure S2.** Inter-individual distances were calculated for WG, WL, and WM participants at three time points over the academic year for (A) unweighted UniFrac and (B) weighted UniFrac distances. Median ± 95% confidence intervals displayed in black. Abbreviations: WG, weight gain (gain of >3% of baseline weight); WL, weight loss (loss of >3% body weight); WM, weight maintenance (less than or equal to ±3% weight change).


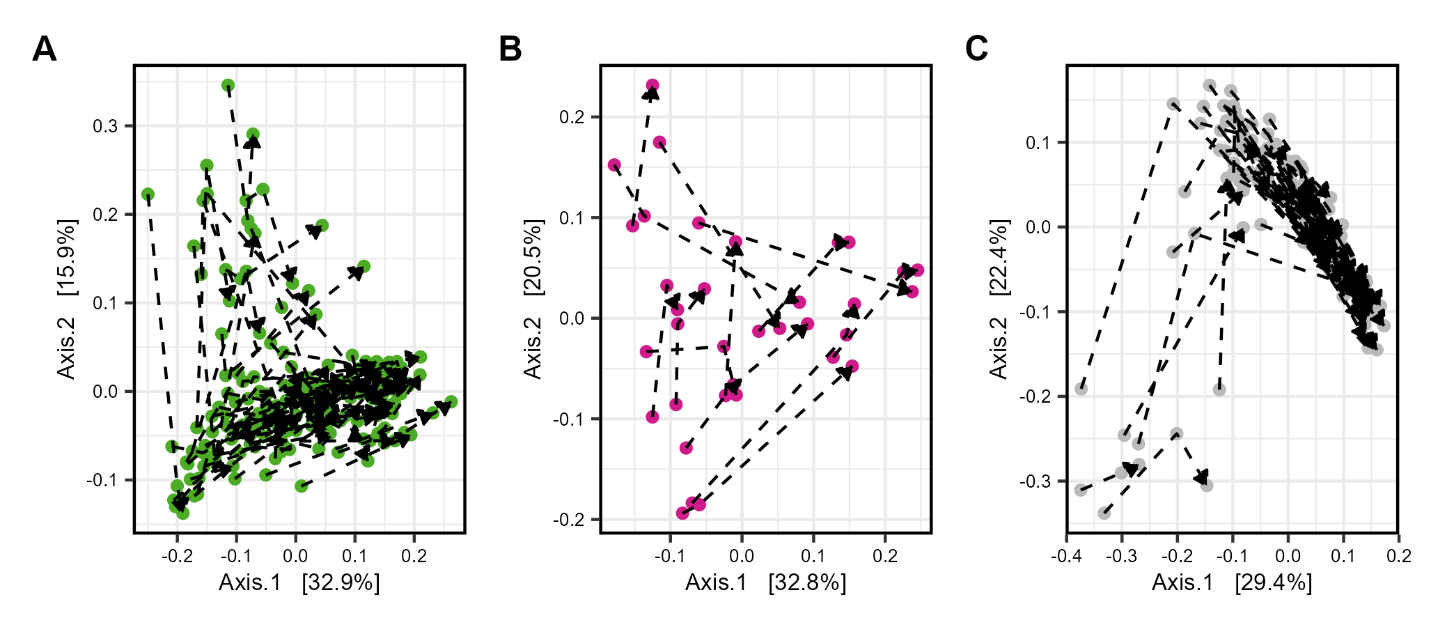


**Figure S3.** Ordination of the first and second principal coordinate axes for weighted UniFrac distances at the genus level for the fecal microbiome of (A) WG participants, (B) WL participants, and (C) WM participants. Dotted lines connect the same individual and point toward the direction of time. Abbreviations: WG, weight gain (gain of >3% of baseline weight); WL, weight loss (loss of >3% body weight); WM, weight maintenance (less than or equal to ±3% weight change).


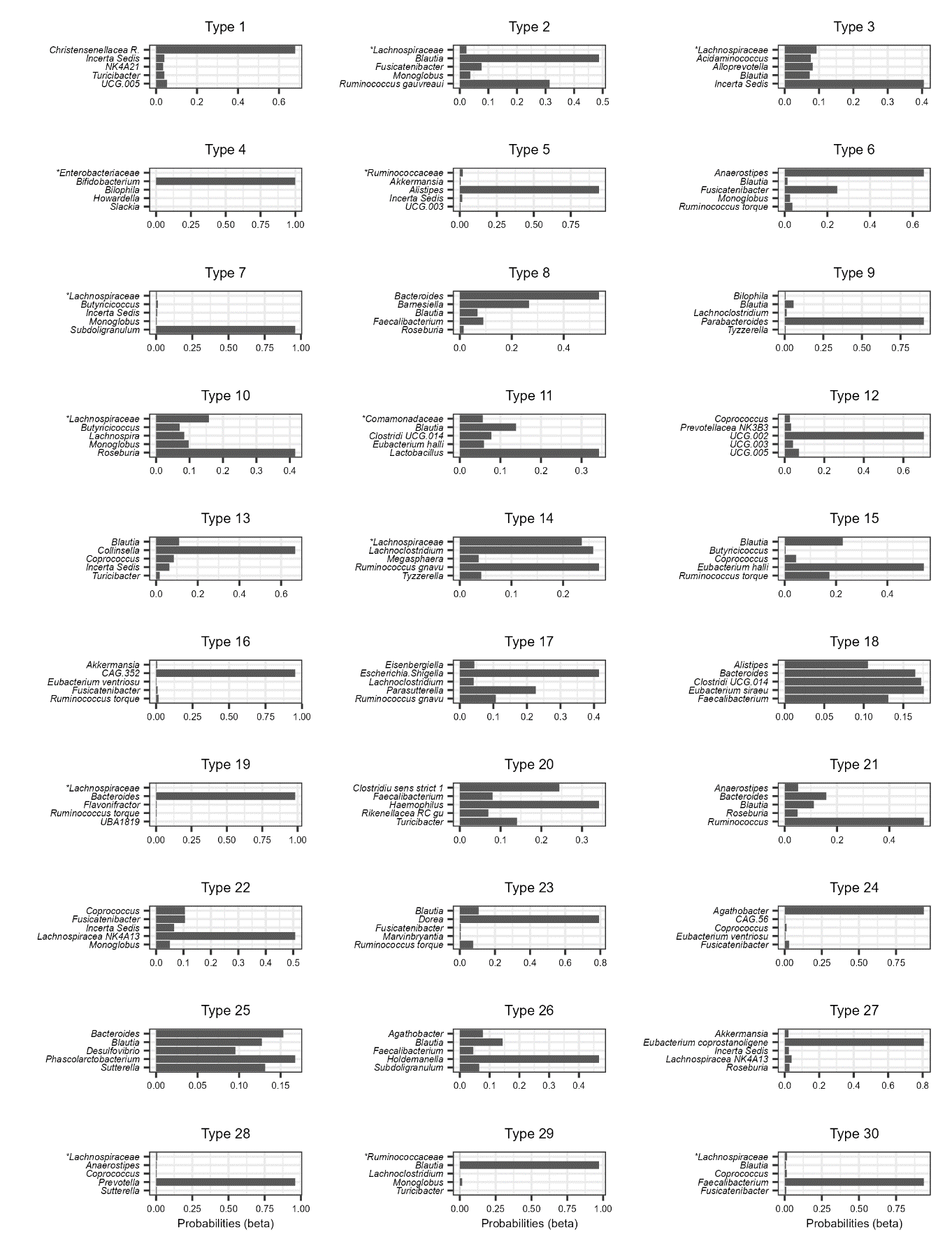


**Figure S4.** The microbiome of 139 college students was defined by 30 community types using latent Dirichlet allocation. Displayed are the top five genera by assigned probability (beta-coefficients) for each community type. Note: The asterisk before a taxonomic feature indicates the family name as the genus was unclassified.


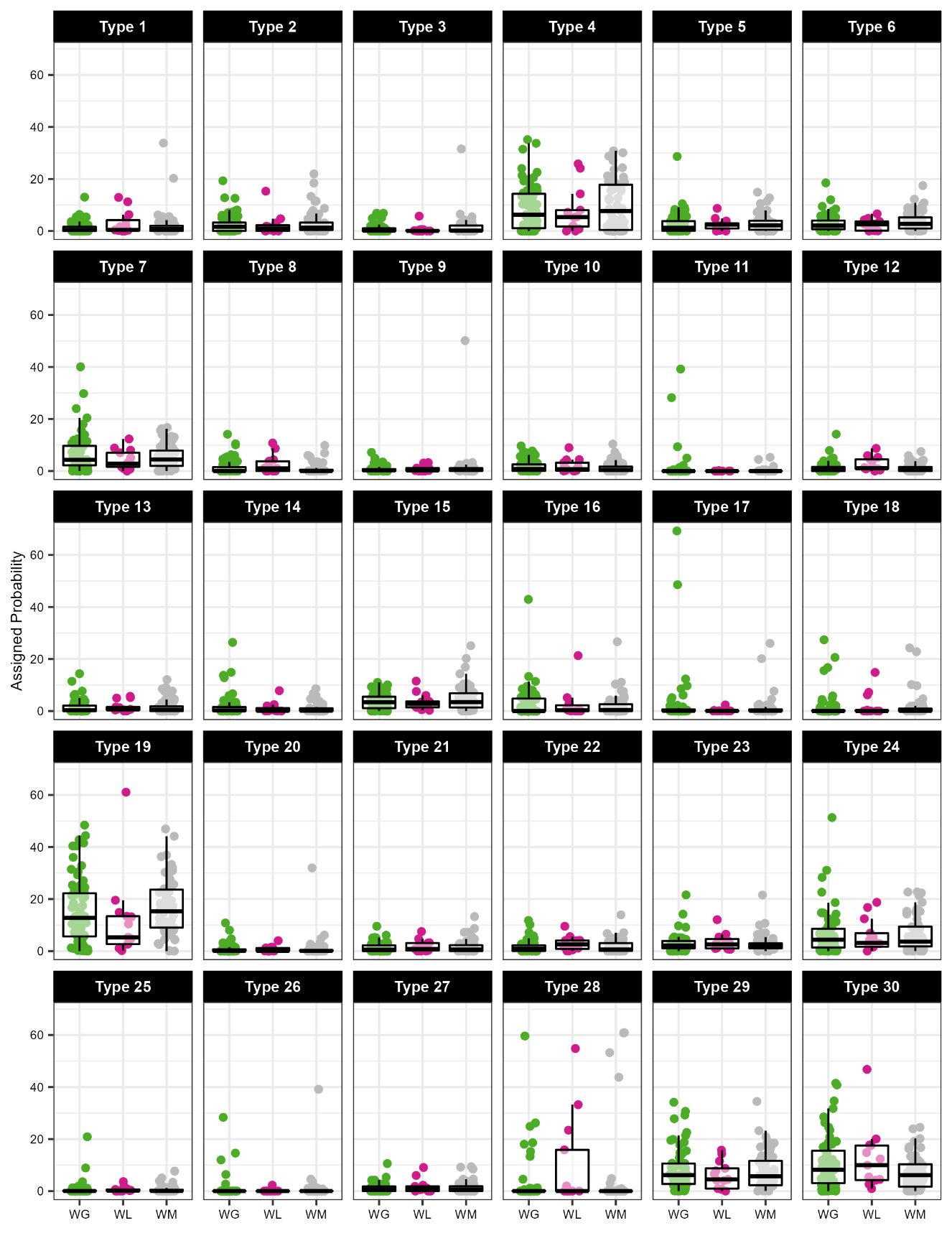


**Figure S5.** Boxplots display the relative abundance of each of the 30 community types at the first sample collection for each of the three weight change trajectories. Community types 12, 22, 28, and 30 had a greater probability of occurrence in WL participants, compared to WG and WM participants, whereas community type 19 had a greater probability of occurrence in WG and WM participants, compared to WL participants (*P.adj* ≤ 0.05). Abbreviations: WG, weight gain (gain of >3% of baseline weight); WL, weight loss (loss of >3% body weight); WM, weight maintenance (less than or equal to ±3% weight change).
